# PD-1^+^ NK cell subsets in high grade serous ovarian cancer: an indicator of disease severity and a target for combined immune-checkpoint blockade

**DOI:** 10.1186/s13046-025-03508-2

**Published:** 2025-08-29

**Authors:** Marco Greppi, Giovanna Tabellini, Ornella Patrizi, Valentina Obino, Matteo Bozzo, Mariangela Rutigliani, Franco Gorlero, Martina Di Luca, Laura Paleari, Ombretta Melaiu, Michele Paudice, Fabrizio Loiacono, Patrizio Castagnola, Valerio Gaetano Vellone, Pascale André, Domenico Mavilio, Gianluca Ubezio, Simona Candiani, Camilla Jandus, Lorenzo Moretta, Andrea De Censi, Daniel Olive, Simona Sivori, Eric Vivier, Fabio Rampinelli, Silvia Parolini, Silvia Pesce, Emanuela Marcenaro

**Affiliations:** 1 Department of Experimental Medicine, University of Genoa, Via G.B. Marsano 10, Genoa, 16132 Italy; 2 Department of Molecular and Translational Medicine, University of Brescia, Brescia, Italy; 3 Department of Earth, Environment and Life Sciences, University of Genoa, Genoa, Italy; 4 Department of Laboratory and Service - Histological and Anatomical Pathology Unit, Ente Ospedaliero (E.O.) Galliera Hospital, Genoa, Italy; 5 Obstetrics and Gynecology Unit, Galliera Hospital, Genoa, 16128 Italy; 6 DINOGMI Department, University of Genoa, Genoa, 16132 Italy; 7 Research, Innovation and HTA, A.Li.Sa. Liguria Health Authority, Genoa, 16121 Italy; 8 Department of Clinical Sciences and Translational Medicine, University of Rome “Tor Vergata”, Rome, Italy; 9 Department of Integrated Diagnostic and Surgical Sciences (DISC), University of Genoa, Genoa, 16132 Italy; 10 Pathology University Unit, IRCCS Ospedale Policlinico San Martino, Genoa, 16132 Italy; 11 IRCCS Ospedale Policlinico San Martino, Genova, Italy; 12 Pathology Unit, IRCCS Istituto Giannina Gaslini, Genoa, 16147 Italy; 13 Innate Pharma Research Laboratories, Innate Pharma, Marseille, France; 14 Unit of Clinical and Experimental Immunology, IRCCS Humanitas Research Hospital, Rozzano, Italy; 15 Department of Medical Biotechnologies and Translational Medicine (BioMeTra), University of Milan, Milan, Italy; 16 Department of Oncology, Ludwig Institute for Cancer Research - University of Lausanne, Lausanne, Switzerland; 17 Bambino Gesù Children’s Hospital, Rome, Italy; 18 Division of Medical Oncology, E.O. Galliera Hospital, Genoa, 16128 Italy; 19 Wolfson Institute of Preventive Medicine, Queen Mary University of London, London, E1 4NS UK; 20 Team Immunity and Cancer, CRCM, Aix Marseille Université, CNRS, INSERM, Institut Paoli-Calmettes, Marseille, France; 21 Aix Marseille Univ, CNRS, INSERM, CIML, Marseille, France; 22 APHM, Hôpital de la Timone, Marseille-Immunopôle, Marseille, France; 23 Department of Obstetrics and Gynecology, Spedali Civili of Brescia, Brescia, Italy

**Keywords:** Immunotherapy, Natural killer cells, Programmed cell death 1 receptor, Tumor infiltrating, Tumor escape, Immune checkpoint, Ovarian cancer

## Abstract

**Background:**

Ovarian cancer (OC) is the fifth leading cause of cancer-related death among women, with High-Grade Serous Ovarian Carcinoma (HGSC) representing the most aggressive and prevalent subtype. Despite promising results in other malignancies, immune checkpoint blockade has shown limited efficacy in HGSC, highlighting the need for alternative immunotherapeutic targets.

**Methods:**

We conducted an integrated analysis combining multiparametric flow cytometry, RNA sequencing, multiplex immunohistochemistry, and functional assays to characterize NK cells isolated from peripheral blood, peritoneal fluid, primary tumor tissue, and metastases in 60 HGSC patients.

**Results:**

We identified a distinct population of PD-1⁺ NK cells enriched in HGSC tumors and metastatic sites but absent in healthy donors. These cells, characterized by a CD56^dim^NKG2A⁺KIR⁺/⁻NKp46⁺CD57^low^ phenotype, displayed impaired cytotoxicity against autologous HGSC targets, correlating with poorer prognosis. Crucially, this dysfunction was reversible upon combined blockade of PD-1/PD-L1, NKG2A, and KIRs. Spatial and molecular profiling revealed that these cells localize within PD-L1⁺/HLA-E⁺ tumor niches, suggesting that immune suppression is spatially and molecularly coordinated. Transcriptomic analysis confirmed their altered functional state and highlighted actionable checkpoint targets.

**Conclusions:**

Our findings uncover a previously underappreciated population of dysfunctional PD-1⁺ NK cells in HGSC and demonstrate that their suppression is reversible through combinatorial checkpoint inhibition. These insights support the development of spatially-informed, NK-targeted immunotherapies for HGSC patients, particularly those resistant to T cell-based strategies.

**Supplementary Information:**

The online version contains supplementary material available at 10.1186/s13046-025-03508-2.

## Graphical Abstract

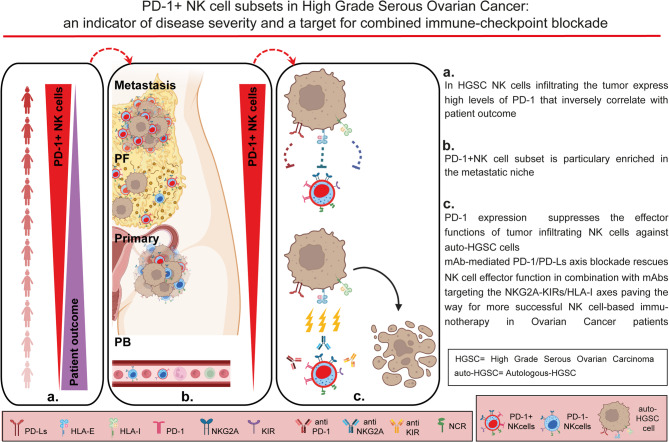

## Background

Ovarian cancer (OC) is the fifth leading cause of cancer-related mortality and the deadliest among female reproductive cancers [1–3]. High-grade serous OC (HGSC), the most common histotype, often remains undiagnosed until advanced stages and is responsible for 70–80% of OC-related deaths [4]. This grim reality is further compounded by nonspecific symptoms and ineffective screening tools. Current treatment for OC typically involves debulking surgery combined with platinum- and taxane-based chemotherapy [5, 6]. Nevertheless, recurrence affects most patients within a few years [7, 8], partly due to the development of ascites/peritoneal fluid (PF), a hallmark of the OC tumor microenvironment (TME), which promotes tumor dissemination throughout the peritoneum and pelvic organs, thereby facilitating metastasis. Thus, improving survival depends on the identification of novel biomarkers for early and accurate diagnosis, as well as the development of innovative and possibly personalized therapeutic strategies [9, 10].

Multiple lines of evidence suggest the presence of Natural Killer (NK) cells within the OC-TME, but with impaired functionality. Indeed, they are characterized by reduced cytolytic activity and inflammatory cytokine production compared to peripheral blood NK cells from the same patient [11–15]. The existence of numerous evasion mechanisms employed by OC-TME to escape NK cell surveillance underscores the pivotal role of these innate cells in OC immunosurveillance [14, 16, 17].

Monoclonal antibodies (mAbs) targeting inhibitory interactions between NK cells and their tumor counterparts, termed immune checkpoints (ICs), have emerged as promising therapeutic avenues [18]. Nonetheless, a thorough understanding of these inhibitory interactions within the OC-TME is still lacking, hindering the discovery of potential therapeutic targets Notably, NK cells are equipped with various ICs, including HLA-I-specific receptors such as killer immunoglobulin receptors (KIRs), NKG2A, and LILRB-1 [19], as well as inducible non-HLA-specific receptors like Programmed Cell Death Protein-1 (PD-1) [15, 20]. These ICs play pivotal roles in maintaining self-tolerance under physiological conditions, including pregnancy, infancy, and adulthood [21, 22]. Remarkably, PD-1 expression in adults is associated with HCMV infection and is predominantly found on fully mature NK cells displaying the CD56^dim^KIR^+^NKG2A^−^CD57^+^NCRs^low^ phenotype. In contrast, in Cord Blood (CB), PD-1 expression on NK cells is independent of HCMV infection and moreover PD-1^+^ NK cells are characterized by a more immature CD56^dim^KIR^+/−^NKG2A^+/−^CD57^−^NCRs^+^ phenotype [21] Interestingly, the expansion of PD-1^+^ NK cell subsets has been observed in patients with hematologic malignancies, certain solid tumors (including OC) [15], or chronic viral infections [23–31].

This study aims to unravel the mechanisms underlying immune dysregulation of NK cells in HGSC patients within the TME, with a specific focus on inhibitory interactions between NK cells and tumors. We performed an extensive analysis of IC expression, primarily PD-1, NKG2A, and KIRs, on NK cells isolated from various anatomical sites, including peripheral blood (PB), PF, primary tumor (T), and metastatic tumor (MT) tissues. Our findings highlight the clinical significance and contributions of these receptors to IC-blockade immunotherapy, providing insights into the design of personalized immunotherapeutic strategies tailored to HGSC patients.

## Methods

### Patients

This study included 60 patients aged between 48 and 82 with HGSC who underwent primary surgery before chemotherapy. The clinical stage of the patients was IIIC, according to FIGO classification. Samples were collected over the course of 4 years and patient outcome was evaluated for the following 5 years. PB derived from voluntary blood donors received at the transfusion center of IRCCS Ospedale Policlinico S. Martino, Genova, Italy, were used as control. Healthy Donors (HD) were selected as donors between the ages of 40 and 60. All biological samples were collected after obtaining informed consent from the donors in accordance with the Declaration of Helsinki. The study was approved by the Ethics Committee of the Region Liguria (Prot. n. 39/2012, number CER Liguria: DB id10125 and Prot. n.127/2022-DB id12223).

### Isolation, culture of human leukocytes

Mononuclear cells were obtained from heparinized PB of HD and HGSC patients and PF of HGSC patients by means of density gradient centrifugation over Ficoll (Sigma, St Louis, MO, USA) and then resuspended in RPMI 1640 supplemented with 2 mmol/L glutamine, 50 µg/mL penicillin, 50 µg/mL streptomycin, and 10% heat-inactivated FCS (Società Prodotti Antibiotici, Milano, Italy). HGSC cells were accessible as single-cell suspensions in PF of patients with advanced disease.

NK cells were isolated from PBMC of PD-1^+^ healthy donors using the NK cell isolation Kit (Milteny Biotec), according to the manufacturer’s instruction. The population of pure NK obtained has a purity greater than 98% defined as CD56^+^/CD3^−^. When necessary, we sorted PD-1^+^ and PD-1^−^ CD56^dim^ NK cells (Beckman Coulter MoFlo Astrios EQ Cell Sorter) defined as CD45^+^CD3^−^CD19^−^CD14^−^CD56^+^(PD-1^+/−^) and the obtained populations had a 98% purity.

To obtain ovarian and metastatic tissue resident cells (lymphocytes as well as cancer cells), accessible as single-cell suspensions we used the gentleMACS Octo dissociator with Heaters (Miltenyi Biotec, Bergisch Gladbach, Germany).

### ELISA

The seropositivity for HCMV in the HD and in the HGSC patients analyzed in this study was detected by using Vironostika anti-CMV III (bioMerieux, Grenoble, France), an ELISA for the detection of total antibody to CMV in human serum.

### Flow cytometry analyses and monoclonal antibodies

The following mAbs were isolated in our laboratory, licensed to the indicated companies, and validated for their specificity: anti–DNAM-1 (KRA236, IgG1), anti-NKp30 (AZ20, IgG1), anti-NKp44 (Z231, IgG1), anti-NKp46 (BAB281, IgG1); anti-NKG2D (BAT221 IgG1), anti–Siglec-7 (QA79, IgG1), anti-Nectin-2 (L14 IgG2A), and anti-PVR (5A10 IgG1). 

For the following mAbs, the specificity has been validated in the corresponding patents or assigned to a cluster in CD workshop (see the indicated link): anti–LILRB-1 (F278, IgG1;www.hcdm.org/index.php?option5com_molecule&cdnumber5CD85J), KIR3DL1/L2-S1 (AZ158, IgG2a; patents/WO2010081890A1?cl5en). The following mAbs were originally isolated at the Laboratoire Immunologie des Tumeurs, CRCM, Marseille-Luminy (France): the purified anti–PD-1 mAb (PD1.3.1.3 clone, IgG2b), the purified anti–PD-L1 (PDL1.3.1 clone, IgG1), the purified anti–PD-L2 (326.35 clone, IgG1); whereas the anti–PD-1–phycoerythrin (PE) or anti–PD-1–allophycocyanin (APC; clone PD1.3.1.3, IgG2b) was purchased from Miltenyi Biotec (Bergisch Gladbach, Germany). Additional mAbs used in this study were as follows: anti-NKG2A–APC and PE-Cy7 (Z199 clone), anti-CD56–PC7 (N901 clone) and CD44-PE were purchased from BeckmanCoulter/Immunotech; anti-CD16–PerCP-Cy5.5 (clone 3G8), anti-KIR2DL2/L3/S2–FITC (CH-L clone), anti-CD107a–PE (anti-LAMP1), CD57 BUV395, CD45 BUV45, CD69 BUV737, CD56 BV480, CD3 PE-Cy5, CD19 PE-Cy5, CD33 PE-Cy5, CD49a BV786, CD103 BV711, CD127 BV650, TIGIT BV605, LAG3 APC-R700, CD90 BV480, Epcam BV786, CD24 BV395, CD45 BV496, CD33 PE-CF594, CD14 PE CF-C94, PD-L1 APCR700, HLA ABC BV650, HLA-E BV421 and 7-Aminoactinomycin D (7AAD), used to stain dead cells from BD Biosciences PharMingen; and anti–KIR2DL1/S1 FITC (11PB6 clone), anti-CD3–VioGreen (BW264/56 clone), anti-CD19–VioGreen (LT19 clone), anti-CD57–VioBlue (TB03 clone), anti-NKG2C–Vio Bright FITC (REA205 clone), NKp30 Biotin, Siglec-7 Biotin, NKp46 PE, CD140a-APC, CD155-PE-Vio770, MICA/MICB PerCP-Vio700 anti-Biotin Vio-615 and FcRBlocking were purchased from Miltenyi Biotec; anti-CD69 (clone FN50, IgG1) was purchased from BioLegend (San Diego), anti-TIM-3 (F38-2E2, IgG1) and anti-LAG-3 (17B4, IgG1) were purchased from Novus Biological; anti-HLA-E (3D12 IgG1) was purchased from Biolegend (San Diego, Calif), anti-B7-H6 (#8750011 IgG1) and anti-TIGIT (#741182, IgG2b) were purchased from R&D. Flow cytofluorimetric (FCM) analyses were performed on a BD FACSVerse or on BD Fortessa flow cytometer (BectonDickinson, Mountain View, Calif) [15], and data analyzed using the FacsSuite (Becton Dickinson, Mountain View, CA) or Flowjo v10 software (TreeStar, Ashland, Ore). We also used FlowJo v10 for visualization of the Uniform Manifold Approximation and Projection (UMAP) [32]. 

**Fig. 1:**
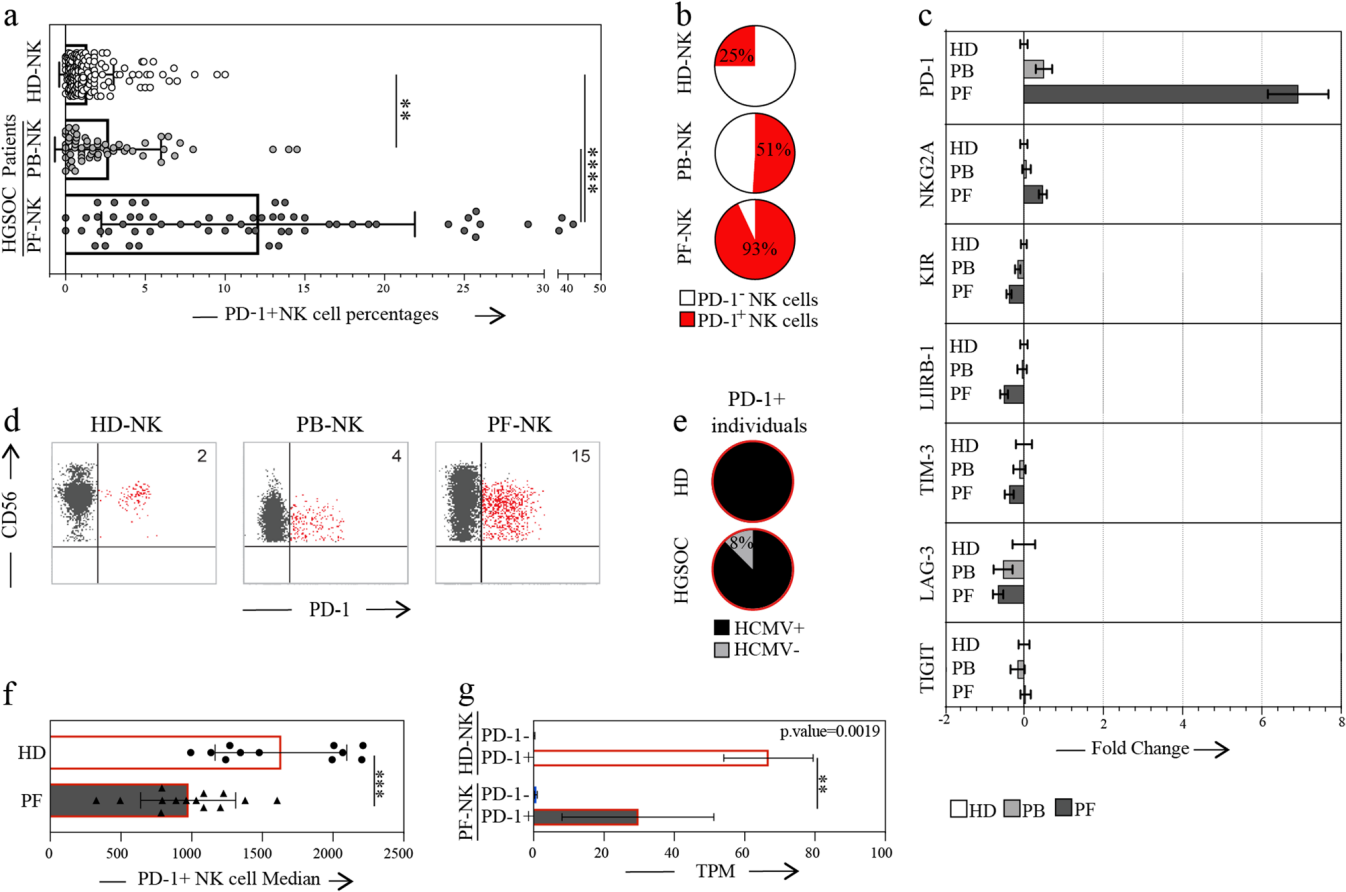
PD-1 expression pattern in NK cells derived from HGSC patients and correlation with patient outcomes. a. Side-by-side expression of the PD-1 immune checkpoint (IC) on NK cells in the peripheral blood of healthy donors (HD-NK, white dots), peripheral blood (PB-NK, light gray dots) and peritoneal fluid (PF-NK, dark gray dots) of HGSC patients (HD n=200, HGSC patients n=60). b. Pie chart showing the ratio of subjects with a PD-1+ NK cell population greater than 1.5% in HD-NK, and in PB-NK and PF-NK of HGSC patients (HD n=200, HGSC patients n=60). c. Fold change of expression of analyzed ICs comparing the mean expression in HD the mean expression of PB and PF from HGSC patients (N>20). d. Expression of PD-1 on NK cells in the PB of a representative HD (HD-NK) and in the PB (PB-NK) and PF (PF-NK) of a representative HGSC patient. PD-1+ NK cells are in red. e. Percentage of seropositivity for HCMV in serum derived from HD (top) and HGSC patients (bottom) expressing PD-1 on NK cells (n=23). f. Different fluorescence intensity of PD-1 staining on PD-1+ cells of HD-NK and PF-NK g. Representation of Transcripts per Million (TPM) of PD-1 transcripts in PD-1+ and PD-1- NK cells of 3 HD and 2 PF samples. Gate strategy: CD45+CD3-CD19-CD14-CD56+(PD-1+/-) **: p< 0.01, ***: p<0.001, ****: p<0.0001

**Fig. 2:**
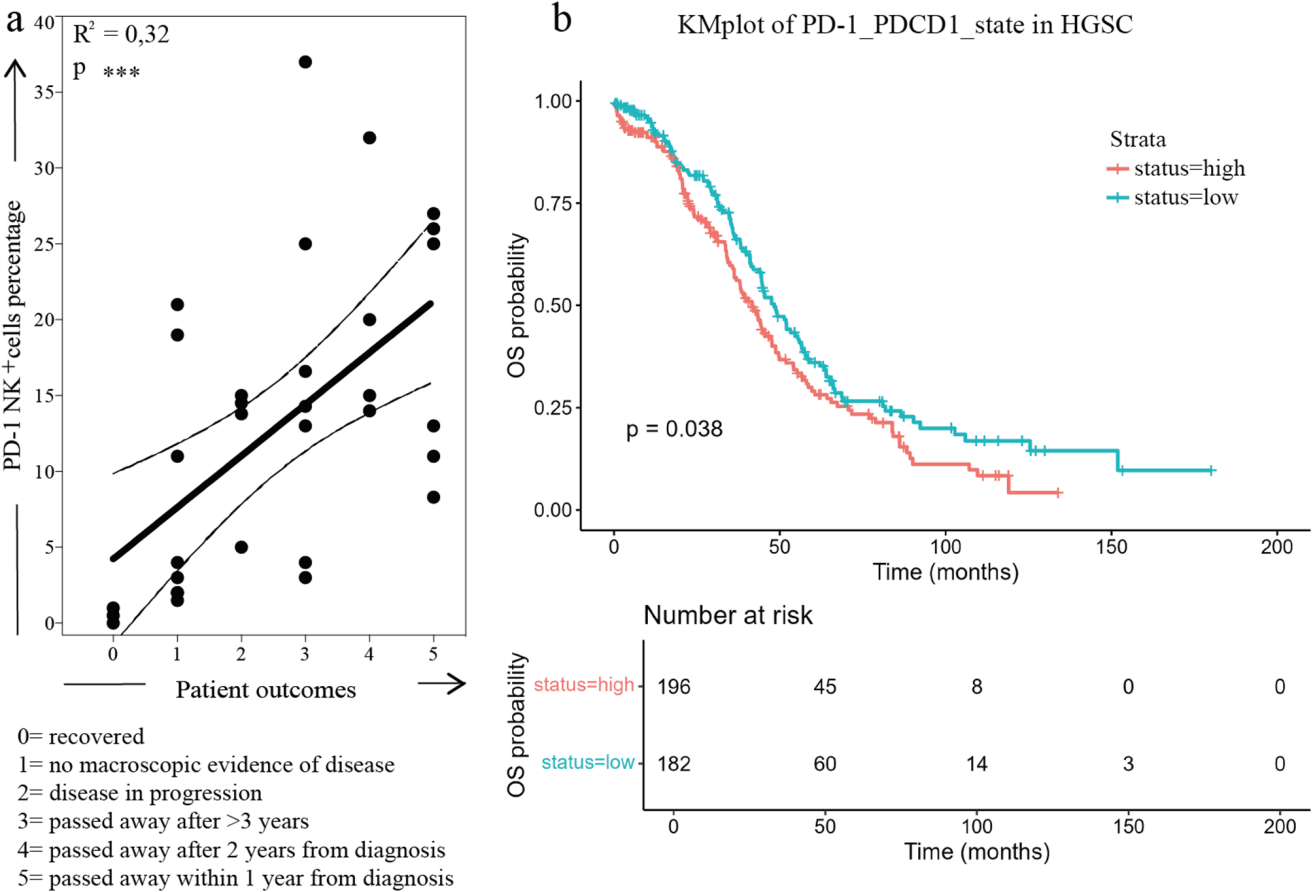
Correlation between PD-1 expression and patient survival.a. Correlation between PD-1 expression on NK cells derived from the PF of HGSC patients analyzed. Patient outcome was categorized in six stages (0-5), ranking from complete recovery to death within one year of diagnosis. b. Kaplan–Meier plots of progression-free survival in patients characterized by high (red) or low (blue) levels of PD-1 transcripts in bulk transcriptomic analysis of HGSC tissue. Data from HGSC patients for whom clinical and gene expression information was available (n=379), downloaded from cBioportal for Cancer. OS: overall survival

### Proliferation assay

The proliferative capability of NK cells was assessed with a CFSE dilution assay by staining PBMC derived from HD and cells derived from PF of HGSOC patients with CFSE before stimulation with rhIL-15 20ng/ml. CFSE staining was assessed immediately after staining and after 3 days. Cells were washed, stained with mAbs indicated in the Figure and analyzed by flow cytometry (BD FACSVerse), cells with the highest CFSE staining after 3 days were defined as non-proliferating, while cells showing a decrease of CFSE intensity were defined as proliferating.

### Degranulation assay

The following target cells were used in this study: the K562 human erythroleukemia cell line, the IGROV and A278 ovarian cell lines, the OVCAR5 metastatic gastrointestinal cancer cell line, originally derived from ascitic fluid, the FcγR^+^P815 murine mastocytoma cell line, and autologous PF-isolated HGSC cells. None of the cell lines used are included in the ICLAC register for mislabeled cell lines v12. For degranulation assay, PB and PF lymphocytes were cultured O.N. in the presence of low doses of rhIL-15 (0.5 ng/mL) and when needed (A278 and IGROV) target cells were cultured O.N. in the presence of IFNγ (50ug/mL) then co-incubated at an E/T ratio of 1:1 in a final volume of 200 µl in round-bottomed 96-well plates at 37 °C and 5% CO2 for 3 h in culture medium supplemented with anti-CD107a-PE mAb. PF-derived HGSC cells were cultured O.N. in the presence of autologous PF. The cocultures with FcγR^+^P815 cell line for reverse antibody-dependent cell cytotoxicity (R-ADCC) were performed in the presence of anti-CD16 mAb (c127, IgG1; Antibodies-Online, Aachen, Germany) in combination or not with anti-KIR2DL2/L3/S2 (GL183 IgG1 clone), anti-KIR2DL1/S1 (11PB6 IgG1 clone), anti-KIR3DL2/3DS1/3DL1 (AZ158 IgG2a clone), anti-NKG2A (Z270 IgG1 clone) and anti-PD-1 (PD1.3.1.3 clone, IgG2b), as indicated in the appropriate figure. The cocultures with the ovarian carcinoma cell lines or OVCAR-5 or autologous HGSC cells were performed in the presence or absence of anti–PDL-1/2 (PD-L1.3.1 clone, IgG1 and 326.35 clone, IgG1), and anti-HLA-I (A6/136, IgM), mAbs as indicated in the appropriate figure (masking mAbs experiments). Cells were washed, stained with mAbs indicated in the Figure and analyzed by flow cytometry (BD FACSVerse).

### Gating strategy

Information on gate strategies is indicated in each figure caption.

### Immunohistochemistry

Immunohistochemical staining was performed on 2 μm thick FFPE serial with an automated IHC staining system (Ventana BenchMark ULTRA, Ventana Medical Systems, Italy). Sequential IHC was performed using an ultraView Universal DAB detection Kit. Heat-induced epitope retrieval was performed using CC1 buffer (standard CC1, Roche Ventana) by boiling for 36 min for CD3, 64 min for RORγt, 8 min for NKp46/NCR1, and 30 min for PD-1. Afterwards, slides were incubated with the following mAbs: anti-CD3 (clone 2GV6, Ventana, prediluted) for 44 min at 37 °C; anti-RORγt (clone 6F3.1, Millipore, dilution 1:20) for 36 min at 37 °C; anti-PD-1 (PD1.3.1.3 clone, IgG2b, dilution 1:50) for 16 min at 37 °C, NKp46/NCR1 (195314 R&D Systems bio-techne catalogue number MAB1850) for 20 min at 37 °C, and then visualized with DAB chromogen. Whole slides were manually digitalized at 10X magnification using an Olympus BX60 and the Microvisioneer Manual WSI software. To assess NKp46 and PD-1 co-localization, two consecutive sections were used, one immunostained for NKp46 and the other for PD-1. Regions of interests were loaded in a stack and manually aligned in Adobe Photoshop CS4. Minor adjustments of brightness contrast were performed. To show co-localization, NKp46 positive cells were digitally highlighted in red and overlapped onto the section immunostained for PD-1, without altering the alignment.

### Multiplex immunohistochemistry

Multiplex immunohistochemistry (mIHC) was performed on 2-µm thick FFPE section using Tyramide SuperBoost Kits from Invitrogen (Thermo Fisher Scientific, Waltham, MA, USA) following the manufacturer’s instructions and using the following fluorochromes: Alexa Fluor 488, Alexa Fluor 546, and Alexa Fluor 647. Antigen retrieval was performed by heating in EDTA buffer (Thermo Fisher Scientific, Waltham, MA, USA) at 95 °C for 20 min. The following antibodies were incubated overnight at 25 °C: CK7 (Abcam EPR17078, 1:50), HLA-E (Proteintech 66530-1-Ig, 1:200), NKG2A (Abcam EPR23737-127, 1:50), NKp46/NCR1 (Bio-techne MAB1850, 1:25), PD-1/PDCD1 (Cell Marque NAT105, 1:50), PD-L1 (Ventana SP263, prediluted). After each round of HRP reaction, antibodies were detached by heating the slides in Citrate buffer pH 6 (Thermo Fisher Scientific, Waltham, MA, USA) with a microwave for 15 min. Nuclei were counterstained with 1 µg/ml Hoechst 33,342. Images were acquired using an inverted Thunder Imager (Leica Microsystems, Wetzlar, Germany).

### RNA sequencing

Total RNA was extracted from both PD-1^+^ and PD-1^−^ FACS-sorted NK cells from three female HD and two HSGOC patients using the miRNeasy Micro Kit (Qiagen) following the manufacturer’s instructions. Paired-end sequencing of polyA-RNAs was performed by Macrogen Europe using the Watchmaker mRNA Library Prep Kit (Watchmaker Genomics) and the Novaseq X (Illumina) platform. Trimmed reads were mapped to *Homo sapiens* hg38 reference genome (annotation NCBI_109.20200522) with HISAT2 [33] and transcripts were assembled using Stringtie [34]. Expression profile was calculated for each sample and transcript/gene as read count, FPKM (fragment per kilobase of transcript per million mapped reads) and TPM (transcripts per kilobase million). Raw data and processed gene counts are available in the GEO data repository (https://www.ncbi.nlm.nih.gov/geo/) under accession number GSE255486. A heatmap of the expression of selected genes was generated from TPMs values using pheatmap in R studio [35].

***To be removed after peer-review: Reviewers are granted anonymous***,*** read-only access to GSE255486 data while they are private using the token (uzyfaqamdhqnxul) at the following link***: https://www.ncbi.nlm.nih.gov/geo/query/acc.cgi?&acc=GSE255486.

### Statistical analysis

The Man Whitney U test was used for evaluating quantitative variables when two non-matching groups are compared, the Wilcoxon matched-pairs signed-rank test was used when evaluating matched pairs, and One-Way ANOVA was used when three or more groups were compared. To calculate the fold change in Fig. 1  each individual value was divided by the average of values for HD-NK. Linear regression was used to model the relationship between PD-1 expression and qualitative properties of the samples assigning numerical value to qualitative data (Life Expectancy Index in Fig. 1 and sample origin in Fig. 8) R^2^, p. value and 95% confidence levels are indicated in the respective figures. Graphic representation and statistical analyses were performed with GraphPad Prism 8 (GraphPad Software, La Jolla, CA, USA).

**Fig. 3:**
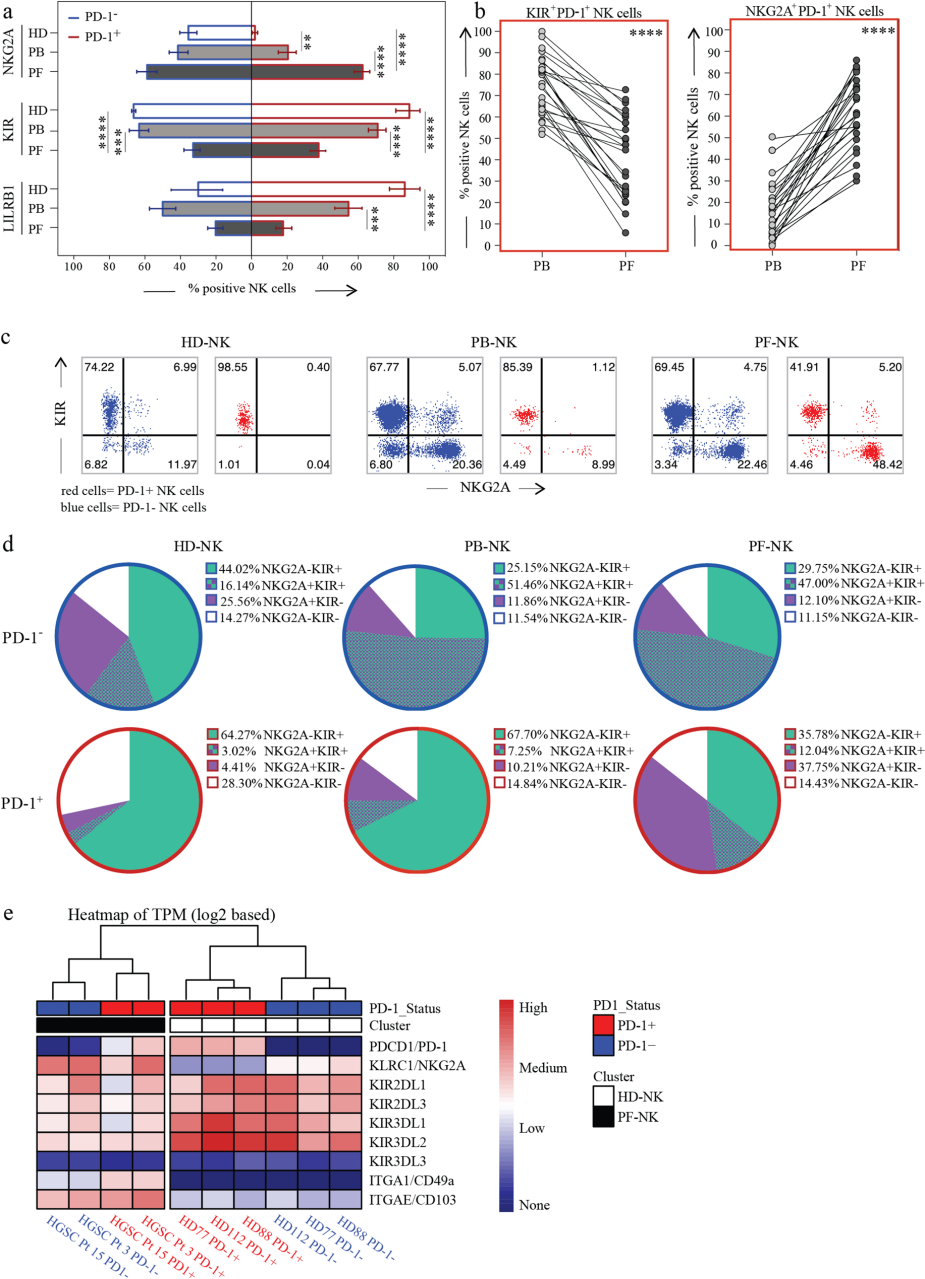
Co-expression of PD-1 with KIRs and NKG2A in HD and in PB and PF of HGSC patients. a. Expression of NKG2A, KIRs and LILRB1 on PD-1-CD56dim NK cells (blue outline) and PD-1+CD56dim NK cells (red outline) in HD-NK (white bars), and NK cells from PB (PB-NK, light gray bars), and PF (PF-NK, dark gray bars) of HGSC patients (n=25). b. Variation in the co-expression of KIRs (left) and NKG2A (right) with PD-1 between PB-NK (light gray dots) and PF-NK (dark gray dots) of HGSC patients. PB and PF samples from the same patient are connected by a black line (n=25). c, d. Representative dot plots (c) and pie charts (d) showing the distribution of KIRs and NKG2A in PD-1-NK cells (blue outline) and PD-1+NK cells (red outline) in HD-NK (left), PB-NK (center), and PF-NK (right) (n=25). e. Heatmap representing relative Transcripts per Million (TPM) of RNA transcripts coding for relevant NK cell markers on sorted PD-1+ (red label) and PD-1- (blue label) NK cells from three HD (white label) and two PF samples (black label). Gate strategy: panels a, c, d: CD45+CD3-CD19-CD14-CD56dimPD-1+/PD-1-; panel b: CD45+CD3-CD19-CD14-CD56dimPD-1+. **: p<0.01, ***: p<0.001, ****: p<0.0001

**Fig. 4:**
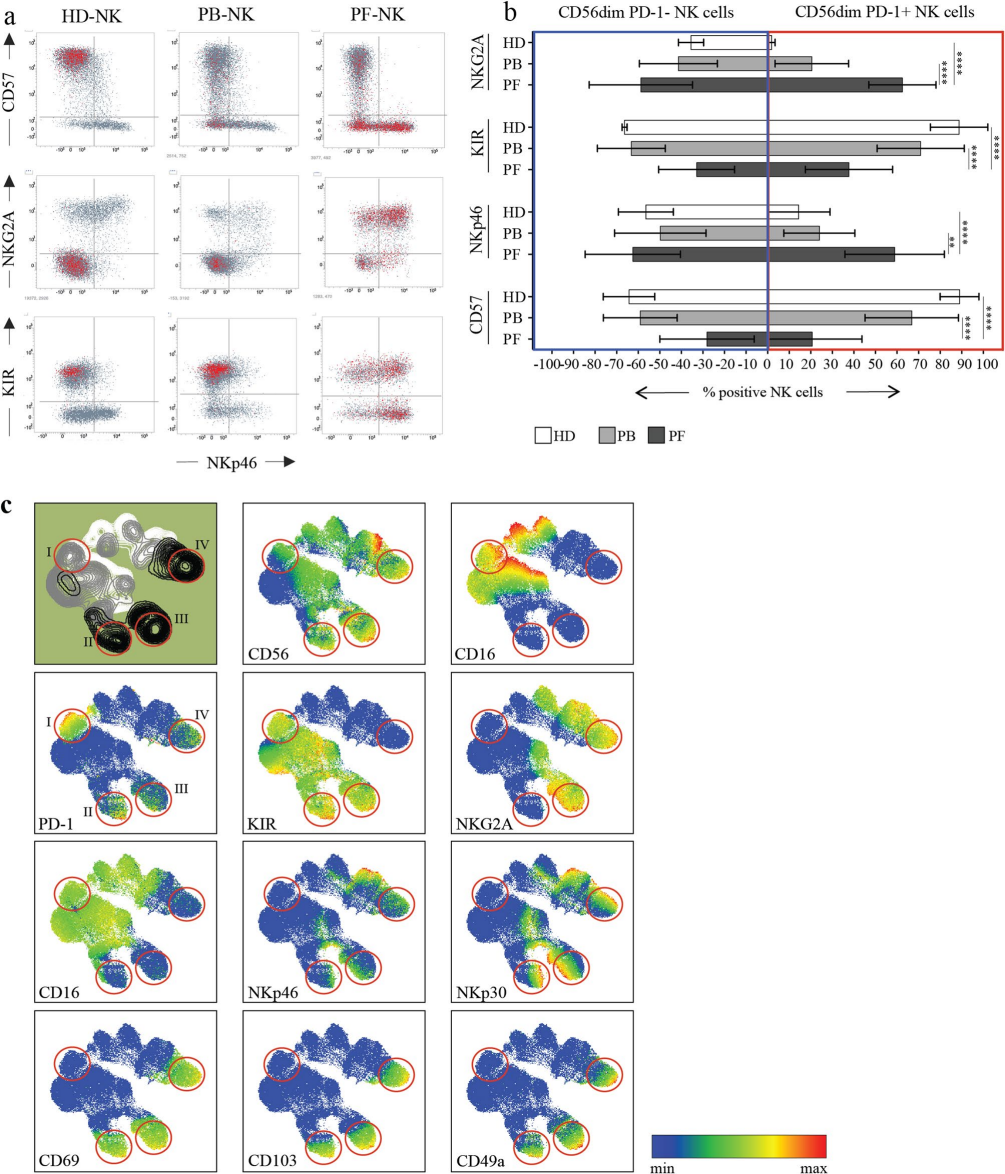
Peculiar PD-1+ NK subsets detectable in HSGOC patients. a. PD-1+ NK cells (red cells) in relation to CD57, NKG2A, KIRs and NKp46 surface expression in a representative HD (HD-NK, left), and a representative HGSC patient (PB-NK, center, and PF-NK, right). b Expression levels of the indicated markers (NKG2A, KIRs, NKp46 and CD57) on PD-1- (blue outline) and PD-1+ NK cell subsets (red outline) in HD (HD-NK, white bars) and HGSC patients (PB-NK, light gray bars and PF-NK, dark gray bars) (n=25). c. UMAP representation of the co-expression of important NK cell markers on NK cells from a representative HD (white area) and the PB (light gray) and PF (dark gray) of a representative HGSC patient, identifying four PD-1+ populations characterized by: I) a KIR+/-NKG2A-CD57+ phenotype characteristic of HD and PB; II) a KIR+NKG2A-CD57- phenotype characteristic of PF; III) a KIR+NKG2A+CD57- phenotype characteristic of PF; IV) a KIR-NKG2A+ CD57- phenotype characteristic of PF. Gate strategy: panel a, c: CD45+CD3-CD19-CD33-CD14-CD127-CD56dim; panel b: CD45+CD3-CD19-CD33-CD14-CD127-CD56dimPD-1+/-. **: p<0.01, ***: p<0.001, ****: p<0.0001

Data from HGSC patients for whom clinical and gene expression information was available (*n* = 379) were downloaded from cBioportal for Cancer Genomics ( https://urlsand.esvalabs.com/?u=https%263%26A%262%26F/www.cbioportal.org%262%26F%26e=ed7a584b%26h=a847d5d9%26f=y%26p=y ). Overall survival curves for groups stratified by the best cut-off value were generated using the Kaplan–-Meier method as implemented in the survival R library. Overall curves were compared using a log-rank test.

## Results

### Tumor-associated NK cells exhibit elevated levels of PD-1 expression significantly associated with a poorer overall prognosis

We performed multiparametric FCM analysis of NK cells from peripheral blood (PB-NK) and peritoneal fluid (PF-NK) in a cohort of 60 high-grade serous ovarian cancer (HGSC) patients. Our analysis revealed a significant accumulation of PD-1^+^ NK cells in both PB (2.66 ± 3.38%) and PF (12.07 ± 9.93%) compartments of HGSC patients, compared to PB-derived NK cells from healthy donors (HD-NK; 1.30 ± 3.79%). Notably, the enrichment was particularly pronounced in the PF, where nearly all patients (93%) displayed PD-1 expression on a distinct NK cell subset, suggesting active induction and/or preferential accumulation of PD-1⁺ NK cells within the tumor microenvironment (TME) (Fig. 1 a-b). While other immune checkpoints were also detected on NK cells, none exhibited such a striking and compartment-specific upregulation as PD-1, making it the most significantly altered checkpoint in the TME (Fig. 1c). As previously described in HD [15], PD-1 expression was largely restricted to the CD56^dim^ NK subset, with negligible expression in CD56^bright^ NK cells (Fig. 1d). Given the established association between PD-1 expression and Human Cytomegalovirus (HCMV) seropositivity in healthy individuals [15], we next investigated this relationship in HGSC patients. In our HD cohort, PD-1⁺ NK cells were detected exclusively in HCMV-seropositive individuals (43.7% of donors), with a frequency of 54.8% among HCMV⁺ subjects, consistent with prior reports. In contrast, although the majority of HGSC patients were HCMV-seropositive, PD-1⁺ NK cells were also clearly detectable in HCMV-negative patients (Fig. 1e), indicating that HCMV is not the sole driver of PD-1 expression in this context. This aligns with recent data showing PD-1 expression on NK cells in newborns independently of HCMV infection [21]. Although elevated levels of cytokines such as IL-12 and IL-18, known to influence NK cell activation and checkpoint expression, were observed in PF [13, 36], no consistent correlation with PD-1 expression was found (data not shown). This indicates that soluble mediators alone are insufficient to fully explain PD-1 upregulation. Instead, PD-1 induction likely results from a combination of tumor-associated factors, including but not limited to soluble mediators and direct tumor–immune cell interactions. Furthermore, the presence of PD-1⁺ NK cells in circulation suggests that NK cells activated within the tumor microenvironment (TME) may recirculate, reflecting a broader systemic immune remodeling in HGSC.

To further investigate the observed differences in PD-1 expression intensity between HD-NK and PF-NK cells (Fig. 1d, f), and considering the potential role of trogocytosis in PD-1 expression on NK cells [37], we performed a transcriptomic analysis of sorted PD-1^+^ and PD-1^−^ NK cells from the PB of 3 HD and the PF of 2 HGSC patients. We detected the presence of PD-1 mRNA in both HD and HGSC PD-1^+^ NK cells, demonstrating that PD-1 is actively produced by NK cells in both healthy and pathological conditions (Fig. 1g). Interestingly, PD-1 mRNA levels were lower in PF-NK cells compared to HD-NK cells, in agreement with the observed differences in PD-1 surface expression (Fig. 1d).

To further validate the clinical significance of PD-1 expression on NK cells, we first analyzed our flow cytometry data derived from patient PF samples, which revealed a significant correlation between elevated PD-1 levels and increased disease severity, with higher expression strongly associated with poor prognosis and survival times not exceeding one year (Fig. 2a).

**Fig. 5:**
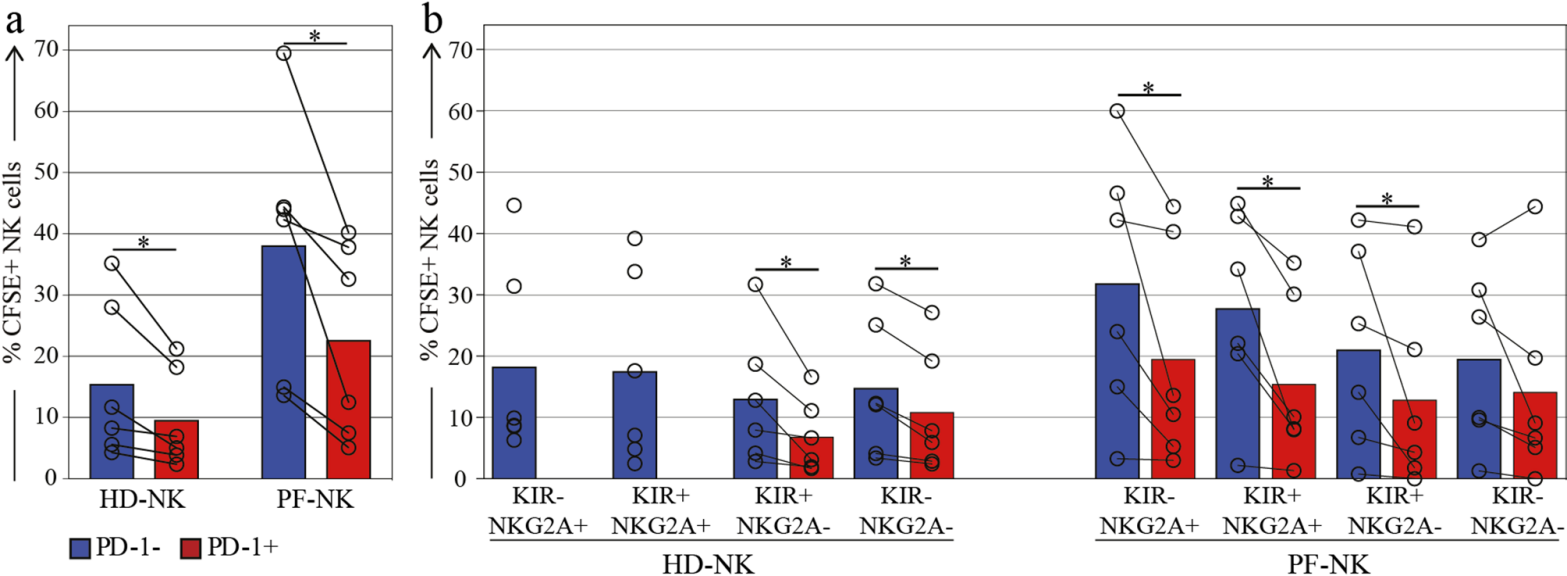
Proliferation assay of NK cells derived from the PF of HGSC patients. a. Proliferation evaluated with a CFSE dilution assay of PD-1- (blue column) and PD-1+ (red column) HD-NK (left) and PF-NK (right) (n=6). b. Proliferation evaluated with a CFSE dilution assay of PD-1- (blue column) and PD-1+ (red column) HD-NK (left) and PF-NK (right) stratified based on co-expression of the classical IC KIR and NKG2A (n=6). Gate strategy: CD45+CD3-CD19-CD14-CD56+

To extend these findings, we then examined a publicly available bulk transcriptomic dataset from a large cohort of HGSC patients (Fig. 2b). This analysis confirmed that elevated PD-1 transcript levels were significantly associated with worse overall survival, thereby corroborating our cellular data. The convergence of both cellular and transcriptomic evidence underscores the robustness of PD-1 as a prognostic marker in HGSC. Notably, this association appeared independent of other clinical variables, suggesting a direct role of NK cell dysfunction in disease progression.

Collectively, these results highlight PD-1 not only as a marker of NK cell dysfunction but also as a critical mediator of immune evasion in HGSC. Such insights provide a strong rationale for the integration of PD-1-targeted therapies into treatment strategies, particularly for patients exhibiting high PD-1 expression on their NK cells.

### Tumor-associated PD-1^+^ NK cells co-express high levels of NKG2A

Given that PD-1 is dramatically upregulated in the HGSC-TME (Fig. 1c), we proceeded with an in-depth analysis of PD-1^+^ NK cells. Interestingly, while in HD there is no co-expression of PD-1 and NKG2A, we observed a limited co-expression of these markers on PB-NK cells and a predominance of NKG2A^+^ cells in the PD-1^+^ subset of PF-NK. In contrast, only a minor fraction of PD-1^+^ PF-NK cells co-expressed KIRs or LILRB1. This is in stark contrast to HD-NK cells, where PD-1 was exclusively co-expressed with KIRs (and LILRB1) but not with NKG2A (Fig. 3a-c) [15].

**Fig. 6:**
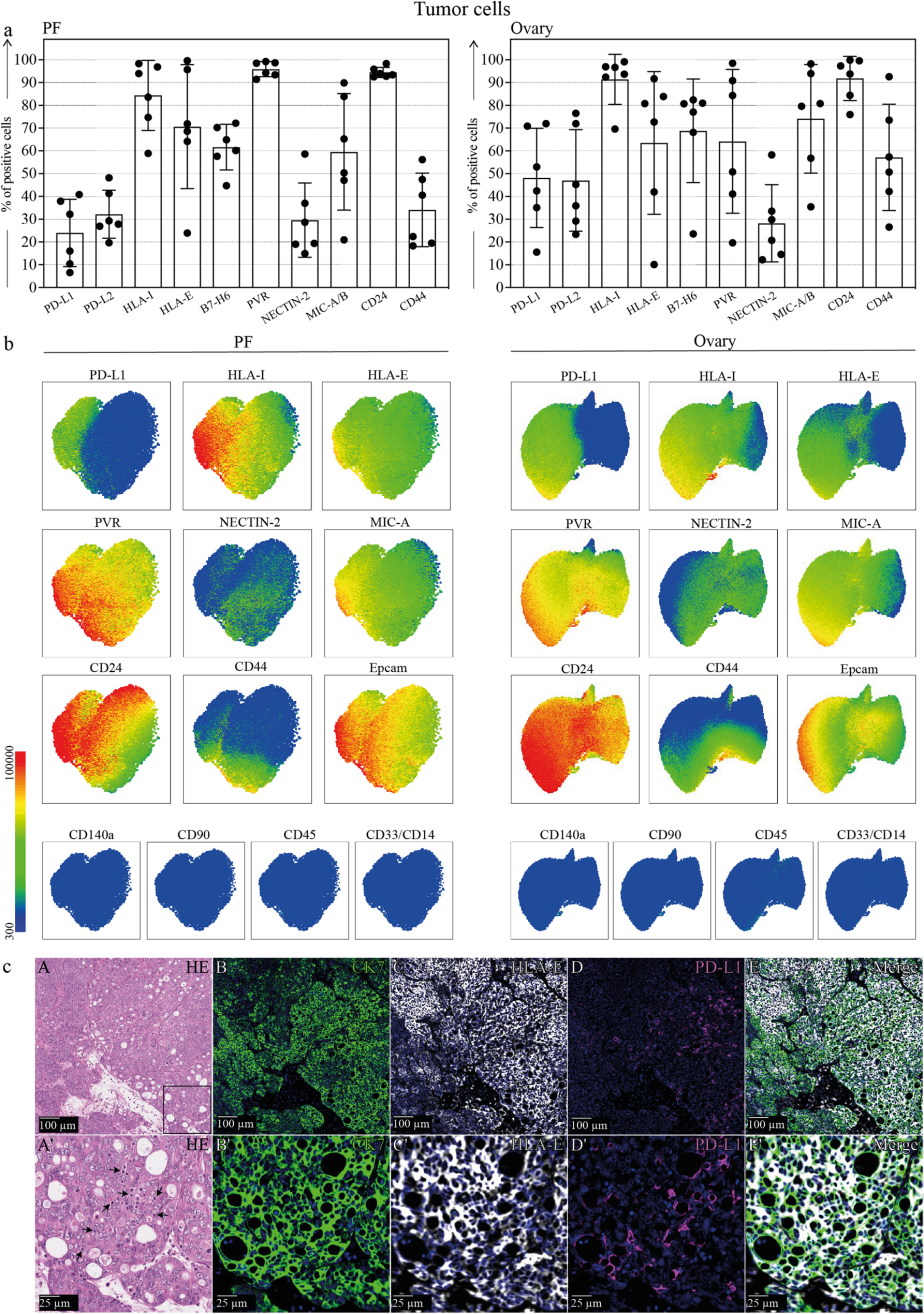
HGSC cells phenotype and ligand expression. a. Expression of inhibitory (HLA-I, HLA-E, PD-L1, PD-L2) and activating (B7-H6, Nectin-2, PVR) ligands on the surface of tumor cells from the PF and the primary tumor of HGSC patients (n=6). b. UMAP representation of most of the analyzed ligands on one representative PF and primary tumor. Gate strategy: 7AAD-CD45-CD14-CD90-CD140a-EPCAM+. c. (A-E) Multiplex immunohistochemical analysis of a representative primitive HGSC tissue showing extensive expression of HLA-E and PD-L1 on CK7+ cancer cells. (A’-E’) Enlargement of the area boxed in A showing that the tumor area is infiltrated by lymphocytes (arrows in A’). Scale bars are indicated in each panel

In summary, the PD-1^+^ NKG2A^+^ NK subset, which was virtually absent in HD-NK cells, was significantly expanded in the PB of HGSC patients, becoming the predominant PD-1^+^ subset in the PF (comprising 37.75% of PD-1^+^ PF-NK). This trend was consistent across all patients analyzed (*n* = 25) (Fig. 3d).

These findings agree with our transcriptomic analyses, which showed that PD-1^+^ HD-NK cells primarily transcribed KIRs, but not NKG2A, whereas PD-1^+^ PF-NK predominantly transcribed NKG2A. In addition, as expected, the tissue residency markers CD49a and CD103 were expressed at high levels exclusively in PF-NK. Based on the expression profiles of immune checkpoints (ICs) and tissue markers, NK cells from HD and HGSC patients clustered into distinct groups (Fig. 3e).

### Tumor-associated PD-1^+^ NK cells exhibit a partially immature phenotype

A more detailed phenotypic analysis revealed that a subset of PD-1^+^ PB-NK and PF-NK not only expressed NKG2A in place of KIRs, but also showed reduced expression of CD57, primarily in the PF. This marker of NK cell maturation was consistently downregulated in both PD-1^+^ and PD-1^−^ PF-NK cells (Fig. 4a, b). Notably, we observed an increased expression of the Natural Cytotoxicity Receptor (NCR) NKp46 in these PD-1^+^NKG2A^+^KIR^–^CD57– NK cells.

Using the UMAP (Uniform Manifold Approximation and Projection) algorithm, we categorized PB-NK and PF-NK cells from an HGSC patient and HD-NK from a healthy donor, both the donor and the patient were selected for their high PD-1 expression (Fig. 4c). The constructed sample in Fig. 4 was generated concatenating an equal number of NK cells derived from PB of an HD and the PB and PF of a patient usong the concatenate function of FlowJoTM v10.8 Software (BD Life Sciences). This sample was represented as a UMAP generated with the following settings: n=15 and min dist=0,5; for color mapping of the UMAP the scale for each marker evaluated was set as follow: the minimum value (corresponding to blue in the scale) was set afyter the negative control and the maximum value (corresponding to red in the scale) was set after highest non outlier value detected for the marker in question. This approach allowed us to better visualize the co-expression of multiple key NK cell markers and assess phenotypic differences between PD-1^+^ NK cells from HDs, PB, and PF compartments in HGSC patients. This analysis shows that PD-1^+^ NK cells from the PB of both HD and patient display a relatively consistent phenotype, which clustered distinctly (area I). This phenotype was characterized by high expression of CD57 and CD16, variable expression of KIRs, low expression of the main NCRs, NKp46 and NKp30, and absence of NKG2A. In contrast, PD-1^+^ NK cells from the PF did not form a distinct cluster. Instead, these cells were interspersed with PD-1^−^ NK cells, suggesting that PD-1 expression in the PF does not correlate with a significantly different phenotype compared to PD-1^−^ cells. We observed notable PD-1 expression in areas characterized by distinct phenotypes: KIR^+^ NKG2A^–^ (area II), KIR^+^ NKG2A^+^ (area III), and KIR^–^ NKG2A^+^ (area IV). Interestingly, regions with higher PD-1 expression overlapped with areas exhibiting low CD16, high NCR expression, and markers associated with NK cell activation (CD69) and tissue residency (CD49a and CD103). Notably, the population with high NCR expression included NK cells from area III, which co-expressed all three inhibitory checkpoints, PD-1, NKG2A, and KIRs, highlighting a highly regulated subset with features of activation and tissue adaptation. These cells are potentially highly cytotoxic due to their elevated NCR expression, but their effector functions appear to be profoundly restrained by the simultaneous engagement of multiple inhibitory pathways.

By comparing the expression of various NK receptors, primarily activating ones, between PD-1⁺ and PD-1⁻ NK cells, we found that PD-1⁺ NK cells from PF displayed elevated levels of the activating receptors NKp46 and NKp30, particularly when compared to PD-1⁺ NK cells from PB and to NK cells from healthy donors. These differences were not observed in the PD-1⁻ subsets. DNAM-1 was markedly downregulated in PF-NK cells, particularly in the PD-1⁺ fraction, but also reduced in PD-1⁻ cells relative to PB and HD controls. No significant differences were found for NKG2D or NKG2C between compartments or PD-1 status. As expected, CD16 was substantially downregulated on PF-derived NK cells regardless of PD-1 expression, while CD57 was significantly reduced, especially in PD-1⁺ PF-NK cells, which typically lack the mature CD57⁺ phenotype seen in PB and HD NK cells. Siglec-7 expression also appeared reduced in PF-derived PD-1⁺ NK cells compared to other compartments. CD69 was strongly upregulated in PF-NK cells, both PD-1⁺ and PD-1⁻, indicating a tissue-resident or activated phenotype [13, 36] (Supplementary Fig. 1).

### Proliferative capacity of PD-1⁺ NK cells in PF

To investigate the proliferative capacity of PF-derived NK cells, we cultured cells for 3 days in the presence of IL-15 and analyzed proliferation by CFSE dilution assay. We compared PD-1⁺ and PD-1⁻ NK cell subsets from the PF of HGSC patients and from PB of HD. PF-NK cells showed overall increased proliferation compared to HD-NK cells (Fig. 5 a). PD-1⁺ cells were consistently able to proliferate, albeit to a lesser extent than their PD-1⁻ counterparts in both HD and HGSC patients. We further stratified NK cells based on co-expression of inhibitory receptors (KIR and/or NKG2A) and observed that PD-1⁺ NK cells proliferated across all subsets, yet consistently less than PD-1⁻ NK cells, regardless of receptor expression. As expected [15], in HD samples, the PD-1⁺ NK cell subset was restricted to more mature KIR⁺ NKG2A⁻ cells, with no detectable PD-1⁺ cells within the NKG2A⁺ subset (Fig. 5b).

**Fig. 7:**
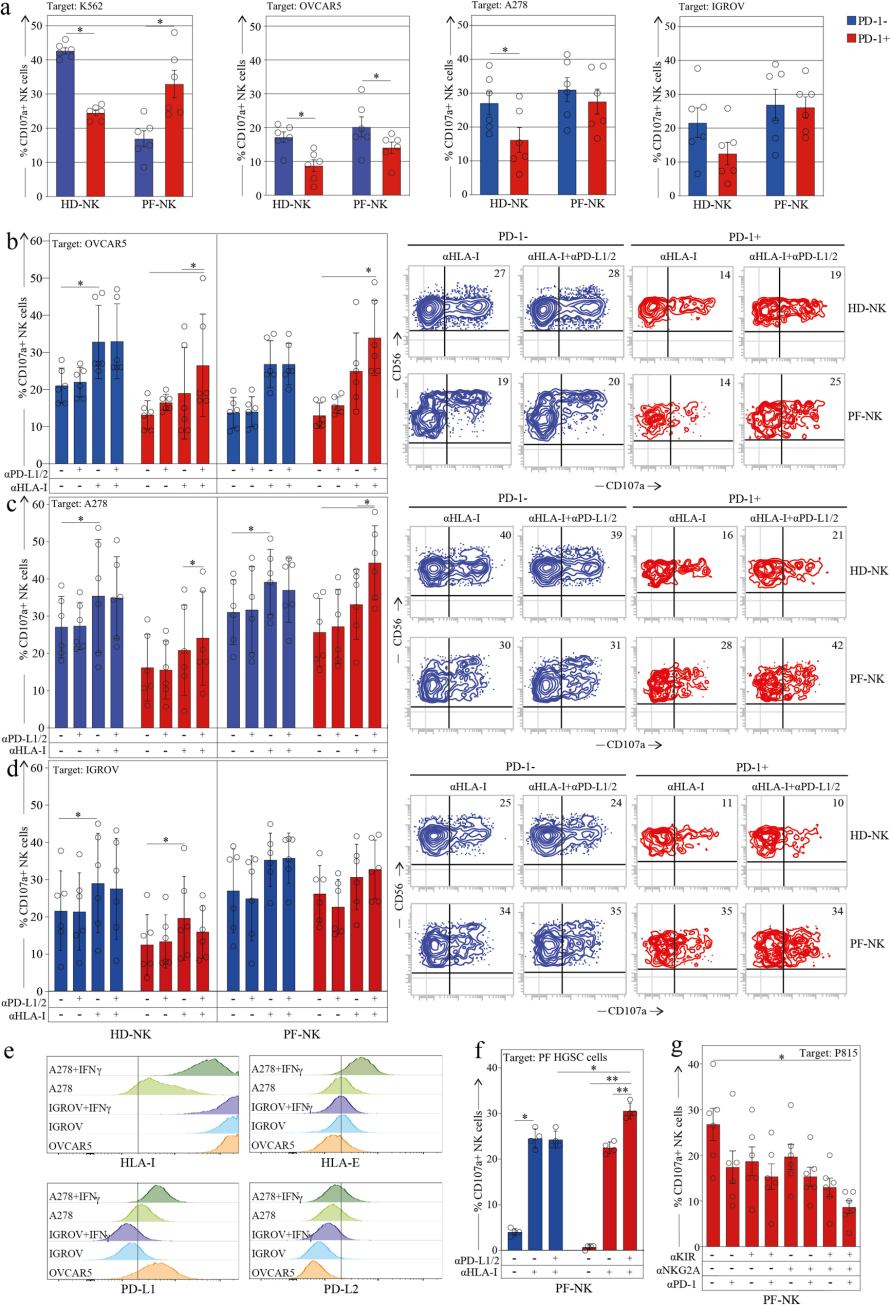
Proliferation assay of NK cells derived from the PF of HGSC patients. (**a**) Proliferation evaluated with a CFSE dilution assay of PD-1- (blue column) and PD-1+ (red column) HD-NK (left) and PF-NK (right) (*n* = 6). (**b**) Proliferation evaluated with a CFSE dilution assay of PD-1- (blue column) and PD-1+ (red column) HD-NK (left) and PF-NK (right) stratified based on co-expression of the classical IC KIR and NKG2A (*n* = 6). Gate strategy: CD45^+^CD3^−^CD19^−^CD14^−^CD56^+^

These findings support the view that PF-derived PD-1⁺ NK cells can undergo local activation and expansion, despite exhibiting a functionally impaired phenotype.

### Phenotypic profiling of HGSC tumor cells in PF and tumor tissue

To accurately characterize tumor cells within the HGSC microenvironment, we applied a stringent gating strategy to exclude immune (CD45⁺, CD33⁺, CD14⁺) and stromal (CD90⁺, CD140a⁺) components. Tumor cells were defined as EpCAM⁺ [38] CD90⁻ [39] CD140a⁻ [40] CD45⁻ CD33⁻ CD14⁻ and further analyzed for the expression of CD24 and CD44, two markers associated with tumor aggressiveness and stem-like features. This multiparametric approach allowed for specific and reliable profiling of epithelial malignant cells in both tumor tissue and PF (Fig. 6a). We next examined the expression of key ligands relevant to NK cell-mediated recognition, focusing on both inhibitory (PD-L1, classical HLA-I, HLA-E) and activating (B7-H6, PVR, Nectin-2, MIC-A/B) ligands for NK cell receptors.

HGSC cells were found to express both PD-L1 and PD-L2 (ligands for PD-1), as well as classical and non-classical HLA class I molecules (ligands for KIRs and NKG2A, respectively). Additionally, HGSC cells expressed ligands for activating NK cell receptors, including B7-H6 (ligand for NKp30), PVR and Nectin-2 (ligands for DNAM-1), and MIC-A/B, ligands for NKG2D (Fig. 6a).

To spatially resolve tumor cell phenotypes, we performed an UMAP algorithm on EpCAM⁺CD90⁻CD140a⁻CD45⁻CD33⁻CD14⁻ cells from both PF and tumor tissue (Fig. 6b). UMAP representations in Fig. 6 were generated from a single sample using the same setting as that used in the Fig. 4. The color mapping was kept consistent between all markers using a logarithmic scale starting at 300 (blue) and ending at 100.000 (red), the minimum value (corresponding to blue in the scale) was set after the negative control and the maximum value (corresponding to red in the scale) was set after the highest non-outlier value detected for all markers.

 The resulting map revealed distinct clustering between compartments. In PF, nearly all tumor cells expressed both HLA-I and HLA-E, confirming their widespread presence in this compartment. Similarly, tumor tissue showed broad expression of these molecules, with only a rare subset of cells lacking both. PD-L1 showed heterogeneous expression across samples, with a higher prevalence in tumor tissue. PVR was consistently expressed, whereas Nectin-2 displayed greater variability, especially in PF. MIC-A/B were broadly detected in both compartments. CD24 was uniformly expressed, while CD44 levels varied across PF and tumor-derived cells.

To further support these findings and spatially validate the expression of key immune checkpoint ligands in situ, we performed Multiplex immunohistochemistry (mIHC) on HGSC tumor sections (Fig. 6c). This analysis confirmed the widespread co-expression of PD-L1 and HLA-E directly on CK7⁺ epithelial cancer cells within the tumor tissue. The staining also revealed the presence of lymphocytic infiltration in the tumor microenvironment, supporting the notion of an immune-infiltrated but immune-suppressed context. These data are consistent with the flow cytometry-based phenotyping and reinforce the relevance of PD-1/PD-L1 and NKG2A/HLA-E axes in mediating immune evasion in HGSC.

### PD1^+^ PF-NK cell subsets are functionally rescued by combined immune checkpoint blockade

We performed functional analyses of PD-1^+^ NK cells from HGSC patients (PF-NK) to evaluate their degranulation capacity, as assessed by surface expression of CD107a following interaction with various target cells, including autologous HGSC cells. To initially assess NK cell functionality, we compared the degranulation capacity of PD-1⁺ and PD-1⁻ CD56^dim^ PF-NK cell subsets in response to K562 erythroleukemia cells [32], K562 cells are widely used as a standard NK-sensitive target due to their lack of HLA-I and PD-L1/PD-L2, and their expression of multiple activating ligands, including B7-H6 (NKp30 ligand) and ligands for NKp46 [41]. PD-1^+^ HD-NK cells (NCRs^low^) [15], exhibited reduced degranulation compared to PD-1^−^ HD-NK cells (NCRs^high^) (Fig. 7a, left) [15]. In contrast, PD-1^+^ PF-NK cells (NCRs^high^) displayed significantly higher cytotoxicity than PD-1^−^ PF-NK cells (NCRs^low^) (Fig. 4). Notably, because K562 cells do not express PD-Ls, the increased cytotoxicity observed in PD-1^+^ PF-NK cells was likely due to the interaction between NCRs, particularly NKp46, which is highly expressed on PD-1^+^ PF-NK cells (see also Fig. 4), and their respective ligands on the target cells.

**Fig. 8:**
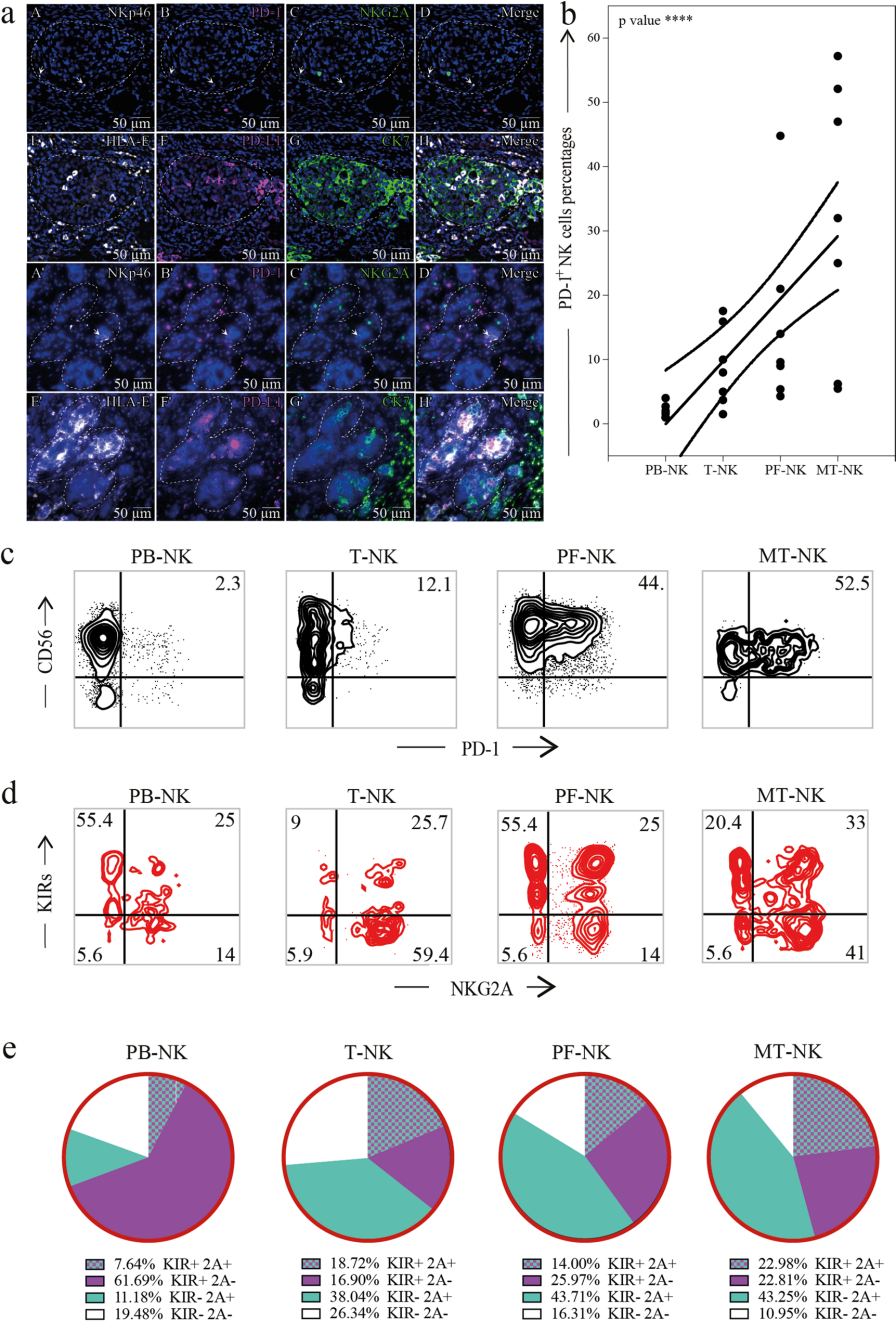
PD-1+ NK cells infiltrate the tumor tissue and are more abundant in metastatic niche. a. Multiplex immunohistochemical analysis of a representative HGSC case showing infiltrating NK cells (NKp46+) expressing PD-1 and NKG2A and expression of PD-L1 and HLA-E on tumor cells (CK7+) in both ovarian and metastatic tissue. (A-H) Primary HGSOC showing co-expression of NKp46 (A,D), PD-1 (B,D), and NKG2A (C,D) on tumor infiltrating lymphocytes and co-expression of HLA-E (E,H), PD-L1 (F,H), and CK7 (G,H) on cancer cells. (A’-H’). Metastatic HGSOC showing co-expression of NKp46 (A’,D’), PD-1 (B’,D’), and NKG2A (C’,D’) on tumor infiltrating lymphocytes and co-expression of HLA-E (E’,H’), PD-L1 (F’,H’), and CK7 (G’,H’) on cancer cells. In all panels, the most prominent tumor cell mass is outlined. Arrows indicate NKp46+/PD-1+/NKG2A NK cells. Inserts in A–D and A’–D’ show cells indicated by yellow arrows at higher magnification. Scale bars are indicated in the Figure. b, c Expression of PD-1 on NK cells in the PB (PB-NK), PF (PF-NK), primary tumor (T-NK), and metastasis (MT-NK) of HGSC patients (n=7) and a representative HGSC patient. 0.0001 p: ****. Linear regression is shown. d, e. Representative dot plots (d) and pie charts (e) showing the distribution of KIRs and NKG2A in PD-1+NK cells in PB (PB-NK), primary tumor (T-NK), the PF (PF-NK), and metastasis (MT-NK) of HGSC patients (n=6). Gate strategy: panel b, c: CD45+CD3-CD19-CD14-CD56dim; panel d: CD45+CD3-CD19-CD14-CD56dim PD-1+

We next assessed the degranulation of the same PF-NK subsets (PD-1^+^ and PD-1^−^ CD56^dim^) following exposure to various HLA-I^+^ target cells, expressing or not PD-Ls molecules. The goal was to determine whether the co-expression of PD-1 alongside constitutively expressed HLA-I-specific inhibitory receptors on PF-NK cells presents an additional barrier to NK cell anti-tumor function.

Although the OVCAR5 cell line does not originate from a primary ovarian tumor, we selected it as the initial in vitro model due to its broad use in NK cell functional studies and several key experimental features. Notably, OVCAR5 expresses all classical HLA class I molecules, which are ligands for inhibitory KIRs, with each donor having a unique set of KIRs specific for distinct HLA-I alleles. It also expresses the non-classical HLA-E molecule, the ligand for NKG2A. These receptors (KIRs and NKG2A) are frequently co-expressed with PD-1 on NK cells derived from HGSC patients. Therefore, the use of OVCAR5 allowed for a standardized and controlled assessment of NK cell cytotoxicity across donors, independent of individual KIR-HLA combinations and licensing profiles (Fig. 7, panel e). Furthermore, the activating NK receptor-ligand expression profile of OVCAR5 closely mirrors that of HGSC tumor samples from our patient cohort (Fig. 6 and data not shown), supporting its utility as a representative and practical model during the initial phase of the study. Nonetheless, to enhance the biological relevance of our findings and address the limitations associated with the use of OVCAR5, we extended our analyses to include two additional epithelial ovarian cancer (EOC) cell lines: A278, which, after stimulation with IFNγ expresses PD-L1, and IGROV-1, which does not. Importantly, these cell lines also exhibit NK receptor-ligand profiles comparable to those detected on primary tumor cells, thus strengthening the translational value of our functional assays. As shown in Fig. 7  (panel e), we performed a side-by-side comparison of classical HLA-I, HLA-E, PD-L1, and PD-L2 expression across all three cell lines, both at baseline and following IFN-γ stimulation. This approach allowed us to investigate how the differential expression of these inhibitory ligands influences NK cell responses, providing a more robust and physiologically relevant validation of the PD-1-dependent inhibitory axis.

As expected, PD-1⁺ PF-NK cells showed reduced degranulation compared to their PD-1⁻ counterparts when co-cultured with PD-Ls⁺ tumor cell lines (OVCAR5 and A278), as illustrated in Fig. 7a (middle panels). This impairment was more pronounced against OVCAR5 cells, which express higher levels of PD-L1, further supporting the role of PD-1/PD-L1 interactions in suppressing NK cell activity. In contrast, PD-1⁺ and PD-1⁻ PF-NK cells displayed comparable degranulation against the PD-Ls⁻ IGROV-1 cell line, indicating that the observed inhibition depends on PD-L expression. While the degranulation of PD-1⁻ PF-NK cells was efficiently restored by anti-HLA-I antibodies alone, PD-1⁺ PF-NK cells required combined blockade of PD-L1/PD-L2 and HLA-I to recover full cytotoxic function against HLA-I⁺, PD-Ls⁺ targets. This suggests that their suppression is not mediated by a single inhibitory axis, but by the cumulative engagement of multiple inhibitory axes. Notably, under dual blockade, PD-1⁺ PF-NK cells exhibited even stronger degranulation than PD-1⁻ cells (Fig. 7b, c), potentially due to their higher expression of activating receptors such as NKp46 (Fig. 4a, b). In contrast, with IGROV targets, which lack PD-Ls, anti-HLA-I alone was sufficient to enhance degranulation, and no additional benefit was observed with combined blockade. This confirms the specific contribution of PD-Ls to the functional suppression of PD-1⁺ NK cells. In a similar experiment, autologous PF-derived HGSC cells were used as targets (Fig. 7e). Once again, PF-NK cells exhibited robust degranulation in response to tumor cells following combined blockade of immune checkpoint ligands with specific monoclonal antibodies. Notably, the impact of the PD-1/PD-Ls axis on suppressing NK cell degranulation was more pronounced in this setting, as indicated by a significant increase in CD107a expression on the NK cell surface following anti-PD-Ls monoclonal antibody treatment (Fig. 7f).

We also evaluated the degranulation of KIR^+^ NKG2A^+^ PD-1^+^ CD56^dim^ PF-NK cells in a reverse antibody-dependent cellular cytotoxicity (R-ADCC) assay using the murine FcγR^+^ P815 mastocytoma cell line (which is negative for PD-L1/PD-L2). These experiments were performed in the presence of anti-CD16 mAb, with or without anti-PD-1, anti-KIR, and/or anti-NKG2A mAbs. The aim was to determine whether NK cell activation could be inhibited by cross-linking CD16 with these inhibitory receptors. mAb-mediated cross-linking of PD-1 led to a significant reduction in PD-1^+^ NK cell degranulation (Fig. 7g). The suppression was even more pronounced when anti-PD-1 was combined with mAbs targeting KIRs or NKG2A, and maximal suppression was observed with the triple blockade of all three ICs. These results clearly demonstrate that the simultaneous expression of multiple ICs provides an additional level of suppression on NK cell anti-tumor responses, which can be functionally rescued by combined IC blockade.

### Multiplex IHC reveals co-localization of PD-1⁺ NK cells with immunosuppressive tumor niches in HGSC

To better characterize the spatial localization and phenotype of PD-1⁺ NK cells in HGSC tissues, including metastatic sites, we employed both multiplex immunohistochemistry (mIHC) and standard IHC staining. The mIHC analysis (Fig. 8a) enabled simultaneous detection of tumor (CK7⁺) and immune (NKp46, PD-1, NKG2A) markers within the same tissue section, allowing precise distinction between tumor and immune components, particularly important in metastatic lesions, where infiltrates are dense and heterogeneous.

Critically, we identified tumor cells co-expressing CK7, HLA-E, and PD-L1, confirming the presence of immunosuppressive ligands within the epithelial tumor compartment. This triple co-expression supports a functional interface between tumor cells and checkpoint-expressing NK cells. Notably, PD-1⁺NKp46⁺NKG2A⁺ lymphocytes were observed in close proximity to these ligand-positive tumor areas, suggesting spatially confined immune suppression.

These findings were further supported by single-marker IHC (Supplementary Figs. 2 and 3), where overlapping NKp46 and PD-1 signals (arrows) indicated the presence of tumor-associated PD-1⁺ NK cells, with higher frequency observed in metastatic cores compared to primary tumors. Flow cytometry (Fig. 8b–e) confirmed an increased proportion of PD-1⁺ NK cells in tumor tissue relative to peripheral blood, with frequent co-expression of NKG2A and KIRs, consistent with a functionally restrained phenotype.

## Discussion

PD‑1 was selected as the central focus of this study due to its marked overexpression on NK cells within the peritoneal fluid (PF) of HGSC patients, significantly higher than in their peripheral blood (PB), and in sharp contrast to healthy donors, whose NK cells rarely express PD‑1 and only within fully mature subsets in HCMV-seropositive individuals [15].

This upregulation correlates with poor prognosis and reduced overall survival, supported by both our patient-derived data and public transcriptomic analyses.

We identified distinct subsets of PD-1⁺ NK cells co-expressing NKG2A and KIRs, selectively enriched within the tumor microenvironment, particularly at metastatic sites. This population is rare in the circulation and virtually absent in healthy individuals. While numerically limited, these checkpoint-positive NK cells consistently accumulate in both tumor tissue and peritoneal fluid, suggesting active recruitment or local expansion. Notably, triple-positive PD-1⁺NKG2A⁺KIR⁺ NK cells represent approximately 13% of tumor-infiltrating PD-1⁺ NK cells overall, but this percentage rises to 19% in primary tumors and 23% in metastatic lesions (Fig. 8), highlighting their progressive enrichment at advanced disease sites. Although they account for ~ 2% of total PF-NK cells and only ~ 0.2% of PB-NK cells, their spatial concentration within tumor compartments underscores a highly localized immunoregulatory role. When including PD-1⁺ NK cells co-expressing at least one additional inhibitory receptor (either NKG2A or KIR), this broader subset exceeds 10% of intratumoral NK cells, compared to ~ 2% in circulation. These findings reveal not only a selective tissue accumulation but also a distinct immunological profile shaped by the tumor microenvironment.

Taken together, these data support the notion that, despite their limited numbers, PD-1⁺ NK cells, and particularly the triple-positive subset, may play a key immunosuppressive and potentially targetable role in HGSC.

Importantly, these NK cells retain high expression of activating receptors such as NKp30 and NKp46, and show proliferative competence, consistent with a functionally restrained rather than terminally exhausted state. Their selective localization, combined with a low frequency in circulation, defines a therapeutic window for combinatorial checkpoint blockade (targeting PD-1, NKG2A, and KIRs) with limited systemic toxicity. Moreover, as NK cells are not restricted by antigen specificity, releasing these inhibitory brakes may unleash broader cytotoxic activity within the TME, enabling a more effective and generalized anti-tumor response.

Using multiplex immunohistochemistry (mIHC), we demonstrated that PD‑1⁺NKG2A⁺ NK cells co-localize with PD‑L1⁺/HLA‑E⁺ tumor regions, revealing a spatially organized immunosuppressive niche where inhibitory ligands and suppressed NK subsets are compartmentalized. This supports a model in which immune suppression in HGSC is both spatially and molecularly orchestrated.

Functionally, although these PD‑1⁺ NK cells can mediate cytotoxicity against standard NK-sensitive targets, they remain ineffective against HGSC cells expressing HLA‑I and PD‑L1/PD‑L2, likely due to concurrent engagement of multiple inhibitory receptors, including PD‑1, NKG2A, and KIRs.

Importantly, PD‑1⁺ NK cells were also detected in the peripheral blood of HCMV-seronegative patients, ruling out CMV infection as the sole driver of PD‑1 expression. Rather, chronic immune activation within the inflammatory microenvironment of HGSC, potentially combined with persistent engagement of activating and inhibitory NK cell ligands on tumor or stromal cells, likely contributes to PD‑1 induction, as observed in other pathological contexts [28, 42].

Despite historical classification of ovarian cancer as poorly immunogenic, accumulating data show that TILs expressing PD‑1 correlate with overall survival and response to ICIs, particularly in BRCA1/2-mutant HGSC tumors with high PD‑L1 expression [11, 43–48]. Although single-agent ICIs have had limited success, combination strategies, including ICIs, vaccines, monoclonal antibodies, miRNAs, and adoptive therapies, show increased promise in improving outcomes [49–51].

Ongoing clinical trials targeting PD‑1, NKG2A (e.g., monalizumab), and KIRs (e.g., IPH2101), alone or in combination, show encouraging efficacy across tumor types (clinicaltrials.gov) [52–55]. Importantly, many TILs simultaneously express multiple inhibitory checkpoints, such as PD-1, KIRs, and NKG2A, reflecting a shared landscape of immune checkpoint regulation between NK and T cells. This overlapping inhibitory profile suggests that multi-target blockade could effectively restore both innate and adaptive immunity in HGSC, representing a compelling therapeutic strategy [49].

In summary, HGSC drives a complex reprogramming of NK cells, locally and systemically, via spatially coordinated inhibitory networks. Understanding these interactions is critical to designing spatially informed, multi-checkpoint immunotherapies, refining patient stratification, and overcoming resistance to T-cell-based strategies, particularly in HLA‑I-low tumors.

## Conclusions

Clinically relevant insights emerging from this study include:

- PD‑1⁺ NK cells are markedly enriched in the peritoneal fluid and tumor tissues of HGSC patients, and are rarely detected in healthy donors, where they are typically restricted to fully mature subsets in HCMV-seropositive individuals.
- This enrichment significantly correlates with reduced overall survival, highlighting their clinical impact and prognostic value.
- The selective accumulation of PD‑1⁺NKG2A⁺KIR⁺ NK cell subsets in tumor-associated compartments, but not in healthy donors, suggests a tumor-restricted therapeutic window.
- Their spatial colocalization with PD‑L1⁺/HLA‑E⁺ tumor microdomains reveals a coordinated immune evasion strategy. The co-occurrence of inhibitory ligands and dysfunctional NK cells supports a spatially organized, molecularly regulated suppression. mIHC-based profiling may improve patient stratification and inform personalized checkpoint-based therapies.
- Despite their inhibitory profile, these NK cells retain activating receptors (NKp30/NKp46) and proliferative capacity, suggesting a suppressed but reversible functional state.
- Combined PD‑1/NKG2A/KIR blockade restores cytotoxicity in vitro, supporting multi-checkpoint strategies.
- Their confinement to tumor/PF sites, especially metastatic lesions, offers a selective immunotherapeutic target with limited off-target risk in HGSC and other HLA-I-low cancers.

## Supplementary Information

Below is the link to the electronic supplementary material.

**Supplementary Material 1**: **Supplementary Fig. 1**: Expression of additional molecules of interest on PD-1 + NK cells from HD and HSGC patients: Histogram representing other molecules of interest expressed on PD-1- NK (blue outline) and PD-1^+^ NK (red outline) cells in the peripheral blood of HD (HD-NK, white bars), peripheral blood (PB-NK, light gray bars), and peritoneal fluid (PF-NK, dark gray bars) of HSGC patients (*n* = 8). Gate strategy: CD45^+^CD3^-^CD19^-^CD14^-^CD56^dim^. *: *p* < 0.05, **: *p* < 0.01, ***: *p* < 0.001, ****: *p* < 0.0001.

**Supplementary Material 2**: **Supplementary Fig. 2**: Immunohistochemical analysis of a HGSC primary tumor showing immune infiltrates expressing PD-1 and NK cell markers.a. Low magnification of primary HGSC in hematoxylin-eosin staining. Scale bar is 150 μm. **b-e**. Enlargement of peritumoral region boxed in **a** showing NKp46^+^ cells in brown (**b**) and red (**c**), PD-1^+^ cells in brown (**d**) and co-expression of NKp46 and PD-1 (**e**, arrows). **f-k**. Enlargement of intratumoral region boxed in **a** showing hematoxylin-eosin staining (**f**), PD-L1^+^ tumoral cells (**g**), CD3^+^ T cells (**h**), PD-1^+^T/NK cells (**i**), NKp46^+^ cells (**j**) and RORγt^+^ cells (**k**). Insert in **k** shows a single RORγt^+^ cell from the positive control specimen (ctr+). NK cells are defined as NKp46^+^RORγt^-^. Antibodies used for immunostaining are indicated in each panel. Scale bar in **b** is 50 μm and is valid for panels **b-k**.

**Supplementary Material 3**: **Supplementary Fig. 3: **Immunohistochemical analysis of a HGSC metastatic tumor showing infiltrates expressing PD-1 and NK cell markers.: a. Low magnification of metastatic HGSC in hematoxylin-eosin staining. Scale bar is 150 μm. **b-e**. Enlargement of peritumoral region boxed in **a** showing NKp46^+^ cells in brown (**b**) and red (**c**), PD-1^+^ cells in brown (**d**) and co-expression of NKp46 and PD-1 (**e**, arrows). **f-k**. Enlargement of intratumoral region boxed in **a** showing hematoxylin-eosin staining (**f**), PD-L1^+^ tumoral cells (**g**), CD3^+^ T cells (**h**), PD-1^+^ T/NK cells (**i**), NKp46^+^ cells (**j**) and RORγt^+^ cells (**k**). Insert in **k** shows a single NKp46^+^ cell from the positive control specimen. NK cells are defined as NKp46^+^RORγt^-^. Antibodies used for immunostaining are indicated in each panel. Scale bar in **b** is 50 μm and is valid for panels **b-k**.

## Data Availability

Raw data and processed gene counts are available in the GEO data repository (https://www.ncbi.nlm.nih.gov/geo/) under accession number GSE255486.

## Abbreviations

7AAD: 7-Aminoactinomycin D
EpCAM: Epithelial cell adhesion molecule
FCM: Flow cytofluorimetric
HD: Healthy donors
HGSC: High-grade serous ovarian carcinoma
IC: Immune checkpoint
LAG − 3: Lymphocyte activation gene 3
mAb: Monoclonal antibody
NCR: Natural cytotoxicity receptors
NK: Natural killer
OC: Ovarian cancer
OS: Overall survival
PB: Peripheral blood
PD-1: Programmed cell death receptor 1
PF: Peritoneal fluid
R-ADCC: Reverse antibody-dependent cellular cytotoxicity
TIGIT: T-cell Ig and ITIM domain
TIL: Tumor-infiltrating lymphocytes
TIM-3: T cell immunoglobulin and mucin-domain containing protein 3
TME: Tumor microenvironment
