# Supplementary figures and images for: PD-1+ NK cell subsets in high grade serous ovarian cancer: an indicator of disease severity and a target for combined immune-checkpoint blockade
Source: J Exp Clin Cancer Res. 2025 Aug 29;44:258. doi: 10.1186/s13046-025-03508-2 (PMC12395815; doi:10.1186/s13046-025-03508-2)

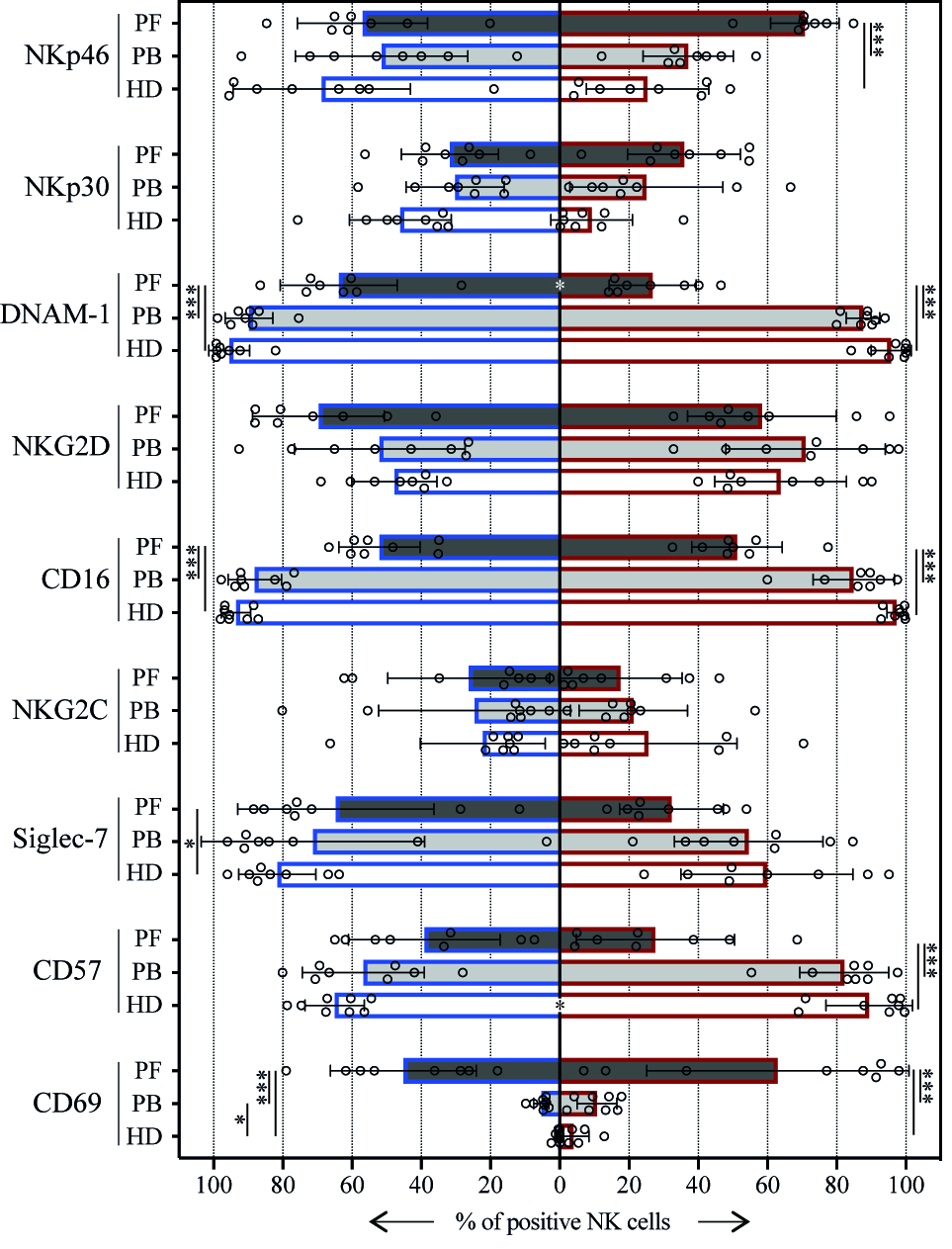

Supplement: Supplementary file 1 — Supplementary Material 1: Supplementary Fig. 1: Expression of additional molecules of interest on PD-1 + NK cells from HD and HSGC patients: Histogram representing other molecules of interest expressed on PD-1- NK (blue outline) and PD-1+ NK (red outline) cells in the peripheral blood of HD (HD-NK, white bars), peripheral blood (PB-NK, light gray bars), and peritoneal fluid (PF-NK, dark gray bars) of HSGC patients (n = 8). Gate strategy: CD45+CD3-CD19-CD14-CD56dim. *: p < 0.05, **: p < 0.01, ***: p < 0.001, ****: p < 0.0001. [file 13046_2025_3508_MOESM1_ESM.tif]

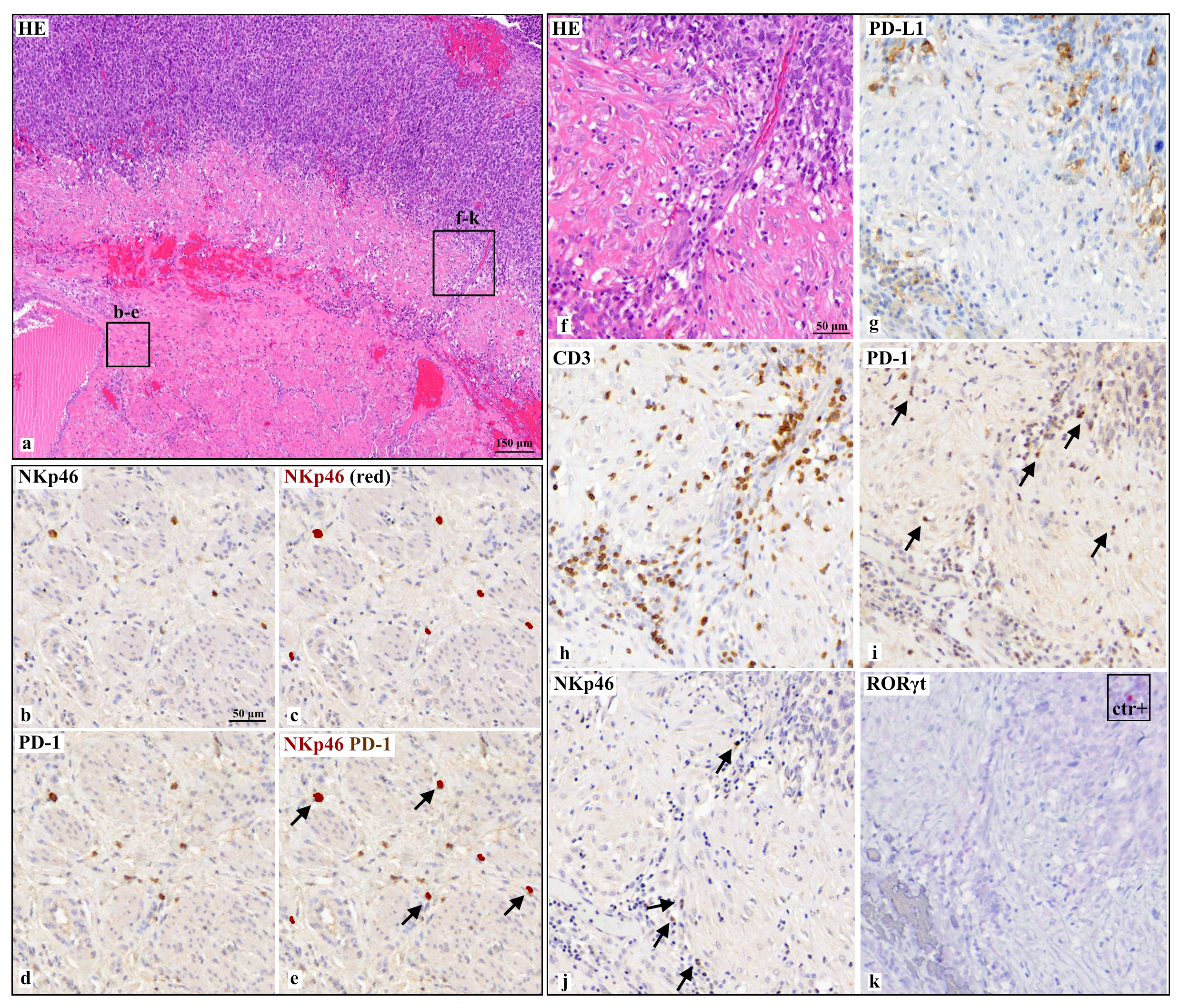

Supplement: Supplementary file 2 — Supplementary Material 2: Supplementary Fig. 2: Immunohistochemical analysis of a HGSC primary tumor showing immune infiltrates expressing PD-1 and NK cell markers.a. Low magnification of primary HGSC in hematoxylin-eosin staining. Scale bar is 150 μm. b-e. Enlargement of peritumoral region boxed in a showing NKp46+ cells in brown (b) and red (c), PD-1+ cells in brown (d) and co-expression of NKp46 and PD-1 (e, arrows). f-k. Enlargement of intratumoral region boxed in a showing hematoxylin-eosin staining (f), PD-L1+ tumoral cells (g), CD3+ T cells (h), PD-1+T/NK cells (i), NKp46+ cells (j) and RORγt+ cells (k). Insert in k shows a single RORγt+ cell from the positive control specimen (ctr+). NK cells are defined as NKp46+RORγt-. Antibodies used for immunostaining are indicated in each panel. Scale bar in b is 50 μm and is valid for panels b-k. [file 13046_2025_3508_MOESM2_ESM.tiff]

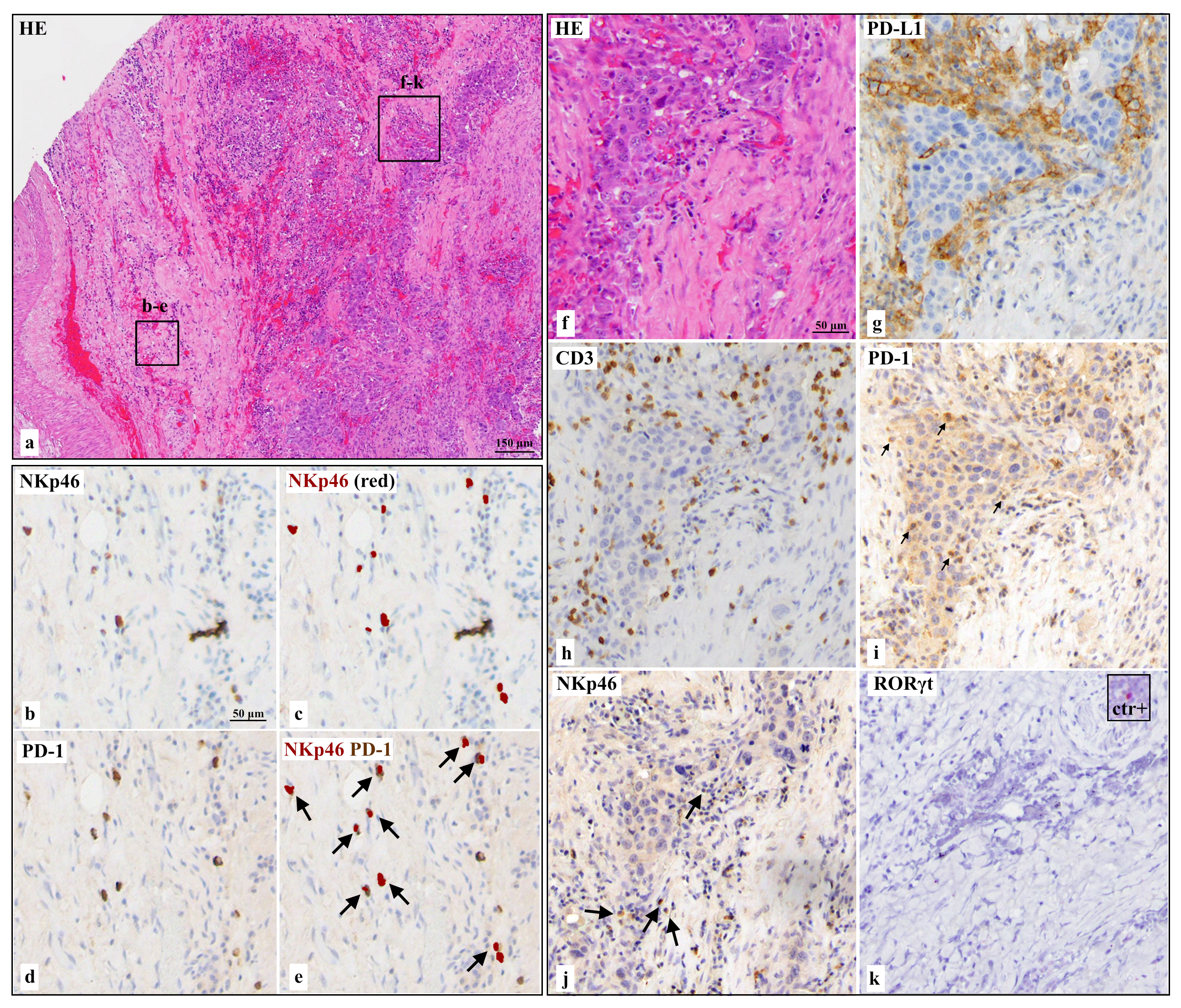

Supplement: Supplementary file 3 — Supplementary Material 3: Supplementary Fig. 3: Immunohistochemical analysis of a HGSC metastatic tumor showing infiltrates expressing PD-1 and NK cell markers.: a. Low magnification of metastatic HGSC in hematoxylin-eosin staining. Scale bar is 150 μm. b-e. Enlargement of peritumoral region boxed in a showing NKp46+ cells in brown (b) and red (c), PD-1+ cells in brown (d) and co-expression of NKp46 and PD-1 (e, arrows). f-k. Enlargement of intratumoral region boxed in a showing hematoxylin-eosin staining (f), PD-L1+ tumoral cells (g), CD3+ T cells (h), PD-1+ T/NK cells (i), NKp46+ cells (j) and RORγt+ cells (k). Insert in k shows a single NKp46+ cell from the positive control specimen. NK cells are defined as NKp46+RORγt-. Antibodies used for immunostaining are indicated in each panel. Scale bar in b is 50 μm and is valid for panels b-k. [file 13046_2025_3508_MOESM3_ESM.tiff]
